# Predicting COVID-19 community infection relative risk with a Dynamic Bayesian Network

**DOI:** 10.3389/fpubh.2022.876691

**Published:** 2022-10-28

**Authors:** Daniel P. Johnson, Vijay Lulla

**Affiliations:** ^1^Department of Geography, Indiana University – Purdue University at Indianapolis, Indianapolis, IN, United States; ^2^Center for Complex Networks and Systems Research, Indiana University, Bloomington, IN, United States

**Keywords:** Dynamic Bayesian Network, Bayesian networks, COVID-19 relative risk, Bayesian hierarchical spatial temporal modeling, social vulnerability, environmental vulnerability, environmental justice, small area studies

## Abstract

As COVID-19 continues to impact the United States and the world at large it is becoming increasingly necessary to develop methods which predict local scale spread of the disease. This is especially important as newer variants of the virus are likely to emerge and threaten community spread. We develop a Dynamic Bayesian Network (DBN) to predict community-level relative risk of COVID-19 infection at the census tract scale in the U.S. state of Indiana. The model incorporates measures of social and environmental vulnerability—including environmental determinants of COVID-19 infection—into a spatial temporal prediction of infection relative risk 1-month into the future. The DBN significantly outperforms five other modeling techniques used for comparison and which are typically applied in spatial epidemiological applications. The logic behind the DBN also makes it very well-suited for spatial-temporal prediction and for “what-if” analysis. The research results also highlight the need for further research using DBN-type approaches that incorporate methods of artificial intelligence into modeling dynamic processes, especially prominent within spatial epidemiologic applications.

## Introduction

The COVID-19 pandemic has caused over 6.4 million deaths worldwide as of summer 2022 and we are only recently beginning to unravel the causative virus's (SARS-CoV-2) spread and patterns of mutation (1, 2). The pandemic has also exposed deficiencies in the developed world—especially with regards to preparedness—to the effects from a highly transmissible infectious disease. It is imperative that the scientific, health, and emergency management communities utilize evidence gained from the COVID-19 health emergency to begin to develop plans to mitigate the next pandemic. Recent research suggests we are likely to experience at least one health emergency that is more pronounced than the COVID-19 pandemic this century (3). Therefore, it is essential we begin the process of furthering the development of preventative public health infrastructure in the face of such a threat. One focus of such an effort should be predictive modeling which forecasts the infection rate of a disease and it's impacts both spatially and temporally. There are numerous models recently published which focus on COVID-19, but it is essential that developed models be dynamic enough to facilitate predictions on a timeframe relevant to public health intervention and response. Artificial intelligence (AI) offers the scientific community the means by which to develop such models and these approaches should be employed in the face of this persistent threat (4–6). By utilizing AI for preparedness and mitigation, public health outreach can be deployed with sufficient spatial-temporal specificity to decrease a disease's impact on a community.

We showcase the development of a model which predicts COVID-19 community relative risk categorically at the census tract-level (small-scale subdivisions of U.S. counties that average 4,000 inhabitants) in the U.S. state of Indiana. The methodology is a hybridized approach using a Bayesian hierarchical spatial-temporal framework to model relative risk and account for spatial and temporal random effects. Then we incorporate a Dynamic Bayesian Network (DBN) to predict risk categories 1 month into the future. The DBN, a type of AI, uses environmental variables and measures of the social environment (i.e., social vulnerability) in its predictive framework. The modeling is presented in a manner allowing rapid replication for other locations and to facilitate further predictive studies that will assist in building predictive public health modeling capacity.

## Background

### Measures of the social and physical environment affecting COVID-19 transmission

There are numerous factors that affect COVID-19 transmission, community immunity, and probability of severe disease. Primarily these are encompassed in the social and physical environment of a particular location at a specific point in time. The social environment is usually more difficult to model dynamically due to data availability and timeliness of collection. Environmental metrics are collected on a more regular basis *via* satellite, surface observation stations, or captured from weather and climate models.

#### Metrics of the social environment

As with many diseases there is a disparity in impact from COVID-19 that primarily lies along socioeconomic and ethnic/racial boundaries (7–9). Most studies conclude that communities with large percentages of those in poverty, being in a minority group, advanced age, and lower educational attainment lead to higher impacts from COVID-19 than communities with lower percentages (7, 10–12). Most of these aspects of a community are captured with indices of social vulnerability. The U.S. Centers for Disease Control and Prevention, maintains the U.S. CDC Social Vulnerability Index (CDC SVI), which includes 15 variables from the U.S. Census Bureau's American Community Survey (ACS), all ranked in order from lower percentages of a variable to higher (13). The 15 variables, after ranking, are grouped into 4 separate domains; socioeconomic status, household composition and disability, minority status and language, and housing and transportation (14). Based on 2018 observations, these variables, domains, and composite CDC SVI score can be mapped at the U.S. County or census tract level for the entire United States. Another technique used to index social vulnerability was developed at the University of South Carolina; the social vulnerability index (SoVI) (15). This technique utilizes a more advanced principal component analysis methodology that reduces the dimensionality of 22 different variables—indicative of social vulnerability—to a number of domains. Each domain is assessed based on the component outputs and scored by the amount of variance explained (16). For example, Indiana using 2014–2018 ACS inputs contained 22 variables which were reduced to 7 domains and one composite SoVI index (17–19).

Community resiliency is defined as the ability to withstand, recover or adjust to an external stressor and is often improperly defined as the opposite of vulnerability. Communities can have high levels of vulnerability and high degrees of resiliency or low levels of resiliency and low vulnerability. The Baseline Resiliency Indicators for Communities (BRIC) was also developed by many of the same researchers that developed SoVI (20, 21). BRIC takes the uncorrelated variables (Pearson's R < 0.70) from over 50 resiliency indicators available from public sources. These variables are Min–Max scaled and the index is scored through a weighted summation. The BRIC is available only at the county-level for the entire United States since many of the variables employed are only accessible at that level of aggregation. Community resiliency, or a lack thereof, has been linked to poor health outcomes in a number of studies (22–25).

#### Metrics of the physical environment

Numerous environmental variables have been shown to be correlated with COVID-19 infection but none more than air temperature and precipitation (11, 26–33). Temperature, measured by the daily average, maximum and minimum, is highly correlated in most studies but the relationship is an inverse one; higher temperatures lead to less COVID-19 rates of infection. The amount of precipitation also leads to lower incidence of COVID-19 (31). Li et al. (1) found a strong inverse relationship between temperature and COVID-19 incidence. Higher temperatures decreased COVID-19 incidence (OR = 0.81) in a study conducted early in the pandemic in the U.S (through April 14, 2020) (34). In a U.S. county level assessment throughout the first year of the pandemic, Johnson et al. (11), found increases of a standard deviation in average 2 m above ground level temperature lowered relative risk of COVID-19 infection by 20.7% and corresponding precipitation increases lowered relative risk by 4.07%. Although these measurements are not the only environmental indicators that are related to COVID-19 (i.e., wind speed, wind direction, humidity) they do represent the variables with the highest established degree of impact.

There have been numerous attempts to create an environmental vulnerability index that is similar conceptually to the aforementioned social vulnerability indices (35–39). In the United States, the Federal Emergency Management Agency (FEMA) has developed the National Risk Index. This index is a composite of an environmental risk score, historic insurance loss estimates, SoVI and BRIC. The environmental risk score is based on 18 individual historic or potential physical hazards; disease/pandemic is not one of the categories (40). The index is available for the entire United States at the county and census tract-level. The NRI was released in later 2020 and as of late 2021 there were no studies available that quantify an association between the index and an external event or events.

### Predicting spatial-temporal distribution of COVID-19 infection

Studies comparing the accuracy of COVID-19 prediction algorithms at fine-scales have found that network-based approaches accounting for spatial interactions between regions, produce less error than alternative methods (41, 42). Sartorius et al. utilize a Bayesian hierarchical susceptible, exposed, infected, removed (SEIR) model to examine spatiotemporal variability of COVID-19 caseloads and deaths in MSOAs (census tract equivalents in the U.K.) in England and forecast hotspots up to 4 weeks in the future (43). Primarily, their findings support the use of Bayesian hierarchical spatial-temporal techniques to successfully unlock important dynamics of the COVID-19 outbreak. In a study using a network model similar to one we develop, Vitale et al. built an object-oriented Bayesian network (OOBN) and used it to infer (simulate) several scenarios regarding critical thresholds for ICU bed occupancy (44). The OOBN was robust enough to model spatial and temporal interactions between variables and to successfully model multiple lockdown scenarios. Their results provide strong evidence for utilizing Bayesian network outputs in policy-making and decision-support environments relative to public health.

Building on the strengths of network-based approaches, the abilities of Bayesian hierarchical spatial-temporal modeling to unravel important space-time dynamics, and the strong inference capabilities of Bayesian networks, naturally lead to the formulation of a hybridized methodology taking advantage of the individual strengths of each approach. This effort utilizes the Bayesian hierarchical spatial-temporal framework for modeling important observed space-time dynamics of COVID-19 infection and the Bayesian network methodology for classification, forecasting and inference.

## Methods

We collected COVID-19 infection counts, measures of social vulnerability and resiliency, and environmental variables to build a predictive model of categorical relative risk for infection from SARS-CoV-2. We utilize a two-tiered approach where, (1) a Bayesian hierarchical spatial-temporal model is fit to smooth relative risk estimates and, (2) a Dynamic Bayesian Network is specified to produce predictions of relative risk on a monthly basis. This modeling is conducted at the census tract-level in the U.S. state of Indiana and is easily adaptable and extensible to other locations. For Bayesian and network computing we employ the bnlearn, gRain, dbnlearn, and INLA packages of the R statistical platform (45–49).

### Data collection

Case counts of COVID-19 infection were collected from the Indiana Network for Patient Care (INPC) database from March 1, 2020 to October 31, 2021 through the Regenstrief Institute in Indianapolis, IN (50, 51). Cases were grouped by month for a total of 20 discrete temporal increments. Environmental data for temperature and precipitation was collected from Google Earth Engine data sources (52–54). We collected both observed and projected data for average maximum daily temperature and precipitation amounts throughout the study time frame. Monthly precipitation and mean maximum temperature projections were acquired from the NASA Earth Exchange Downscaled Climate Projections (NEX-DCP30) which is a general circulation coupled model (55, 56). We used an average of the Representative Concentration Pathways (RCPs) outputs and utilized these estimates for the month for which predictions are to be made. Importantly, these data are climate projections and not actual weather observations. The general purpose of the dataset is to provide high resolution projections useful for the evaluation of climate change impacts at fine-scales (56, 57). Therefore, we felt this was an essential dataset to use for our monthly predictions. It is also ideally suited to simulate situations where we have no observed values for validation. Observed average daily maximum temperature and the sum of precipitation was calculated from the North American Land Data Assimilation System (NLDAS) and collated monthly (58). Zonal average monthly high temperature and zonal monthly sum for precipitation were calculated for each census tract in the U.S. state of Indiana (1,503 total census tracts).

Measures of social vulnerability were collected from the U.S. CDC SVI and SoVI. The U.S. CDC SVI is available through the U.S. CDC website and we acquired SoVI using both the Vulnerability Mapping Analysis Platform (VMAP) (http://vulnerabilitymap.org) and available GitHub scripts (13, 17–19). Some discrepancies were observed between the two SoVI datasets and for the purposes of this research, we utilized the proprietary VMAP variables to maintain consistency with others using these products. The SoVI dataset only contained 1488 census tracts for Indiana and we used an average SoVI score for the 15 tracts present in our original dataset that were absent from SoVI. The U.S. Federal Emergency Management Agency (FEMA) National Risk Index (NRI) is available through the FEMA website and includes the NRI score itself along with the BRiC variable and a 2010–2014 version of SoVI; compared to the 2015–2018 SoVI used in this study. We used only the NRI and BRIC score from the NRI dataset (40).

### Bayesian and Dynamic Bayesian Networks

Bayesian Networks (BN) are graphical models representing variables along with their conditional dependencies. BNs are represented with directed acyclic graphs (DAG) where directional arcs link dependency between variables or nodes. Joint probability functions are determined by the chain rule of probability within the arc and node representation. Conditional probability tables allow evaluation of each node in the network and their interactions. Nodes which have parents are conditional and those without are marginal. BNs have been shown to be highly adaptable and consistently accurate in a number of applications (44, 59–68). One of the simplest BNs is the naïve Bayes classifier, naïve Bayesian network, or “idiot” Bayes, which has proven to be a valuable, accurate, and robust classification technique (69).

**Figure 1:**
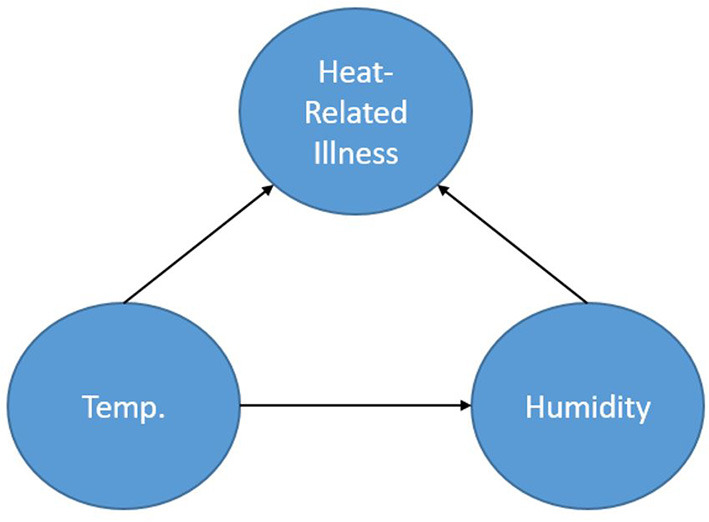
A simple Bayesian network.

Figure 1 shows a simple BN used to represent a calculation for the probability of heat-related illness based on temperature and humidity. The representation shows the node Heat-Related Illness dependent upon the nodes Temperature and Humidity. The node, Humidity (in this case absolute), is dependent on Temperature and Temperature is dependent on neither Humidity nor Heat-Related Illness. By obtaining a dataset consisting of temperature, humidity and heat-related illness rates for a location, one could calculate the parameters of this specified network thereby creating conditional probability tables based on the data and begin inference. Furthermore, if one does not have access to the data but to some descriptors of the distributions of the data for each node, prior probabilities could be specified for the calculation of the parameters. As a simplistic example, one could query the network asking: “What is the probability of heat-related illness when the temperature is in excess of 90 degrees F and absolute humidity exceeds 30 g/m^3^?”.

**Figure 2:**
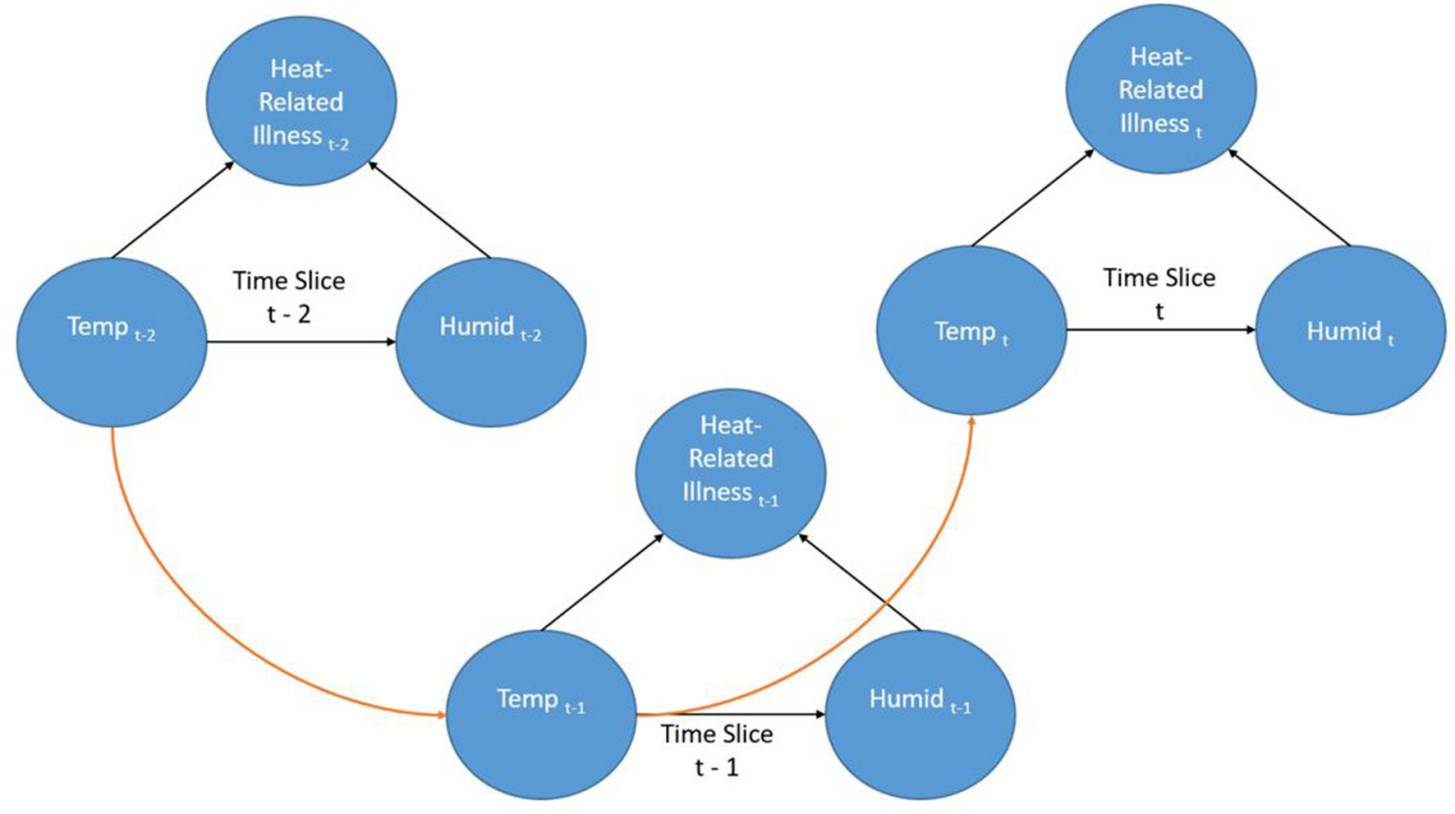
A simple Dynamic Bayesian Network.

Dynamic Bayesian Networks (DBN) are extensions of BNs (an “unrolled” DBN is a BN) that are often recursive in form and adaptable to dynamic forms of data such as a time series. Time is represented through “time slices” where dynamic nodes are established based on their temporal relationships. Often in a DBN architecture a time-slice is dependent on values of the previous slice representing a first order Markov process; although other orders are possible. This creates a recursive pattern within the DAG where the structure of the nodes is consistent between time steps. Even though the contemporaneous network structure can change from one step to another this is often not the case and the structure is entirely recursive. Figure 2 shows a very simple DBN which is an extension of the BN in Figure 1 and demonstrates the recursive pattern. This example network would represent heat-related illness through discrete time steps. In this representation, there are three time slices where temperature is linked to humidity and heat-related illness within the time slice (contemporaneous) but the temperature at time slice t is also dependent on the temperature from the previous time slice (t-1) and so forth. Not only can such a dynamic structure model frequently encountered real-world situations, it can also represent incremental steps in actual decision-making processes.

Another key benefit of the BN approach is that it is not a “black box” which is a common criticism of other network-based approaches (i.e., artificial neural networks, deep-learning convolutional neural networks). One can determine the form of the problem being modeled through decomposition of the network and the conditional probability tables. For example, from the BN presented in Figure 1 we can factorize the probabilities as:


$$\begin{array}{l} \mathrm{Pr}\left(HRI,\;Humidity,\;Temp\right)=\mathrm{Pr}\left(HRI|Humidity,\;Temp\right)\\ \;\mathrm{Pr}\left(Humidity|Temp\right)\mathrm{Pr}(Temp) \end{array}$$

This level of detailed access to the conditional probability tables in the network allow one to determine strengths of association; this is a much more difficult and involved process in many artificial neural network and deep learning approaches.

### Relative risk estimation

Relative risk in a spatial-temporal study is a metric that defines the “relative” risk of a location compared to the overall risk in the study area at a discrete time interval (area_i_ at time_t_). When modeling relative risk in small area studies, spatial Bayesian hierarchical techniques have shown the greatest flexibility and accuracy (70, 71). When dealing with disease counts, aggregated by small areas, there can be large fluctuations in background population from one area to another (72, 73). As an example, without incorporating smoothing techniques, the relative risk in an area with a small population can fluctuate dramatically depending on the number of infected. These are usually reported as a standardized morbidity rate or SMR; which is simply the observed number of infections/expected number of infections. If a neighboring location contains a varying number of cases and a very low population through time, the SMR can markedly fluctuate. Bayesian hierarchical spatial and spatial-temporal modeling, with the inclusion of proper terms, accounts for potential large fluctuations by borrowing information both globally and locally within the defined study area to provide a smooth model absent problematic variations (74). Furthermore, the inclusion of spatial, temporal, and spatial-temporal random effects can account for variables not present in the model, which can lead to numerous complications (74–77). These and other benefits of Bayesian hierarchical modeling enable its usage in a wide array of applications, especially in spatial and spatial-temporal settings.

In this study, in addition to using the SMR, we calculated relative risk using the counts of COVID-19 infection modeled with a Negative Binomial distribution.


$$\begin{array}{l} Y_{it}\;\sim \;NB\left(E_{it}\theta_{it}\right),\;i=1,\ldots ,1503,\;j=1,\ldots ,20 \end{array}$$

*Y*_*it*_ is the number of infected individuals at area_i_ & time_t_. *E*_*it*_ is the expected number of infected based on the total population used as an offset and θ_*it*_ is the relative risk. We included spatial, temporal, and spatial-temporal interaction terms using a scaled Besag, York, Mollié model (BYM2) with a penalized complexity prior (74, 78, 79).


$$\begin{array}{l} \log\left(\theta_{it}\right)=\;d_{i}\beta +\frac{1}{\sqrt{\tau }}(\sqrt{1-\;\varphi }\;S_{*}+\;\sqrt{\varphi }U_{*})+\;\gamma_{t}+\omega_{t}\\ \;+\;\delta_{it} \end{array}$$

*d*_*i*_β is a vector containing the coefficients, S_*_ and U_*_ are, respectively, the spatially structured and unstructured random effects of the BYM model; scaled to follow the BYM2 specification (74, 78, 79). γ_*t*_ &* ω*_*t*_, correspondingly represent the temporally structured and temporally unstructured random effects. γ_*t*_, is modeled as a conditional autoregressive random walk of order one (RW1), and ω_*t*_ is modeled as a Gaussian exchangeable IID ($\omega_{t}\;\sim \;Normal(0,\frac{1}{\tau_{\omega }}))$. The space-time interaction component δ_*it*_, also modeled as IID, represents a parameter vector that varies jointly through space and time. This allows for deviations from the space and time structure expressing both dynamic spatial changes from one time frame to another and active temporal patterns from one area to another (76). We examined all spatial-temporal interactions specified by Knorr-Held and found Type I specification to best fit the data based on Deviance Information Criteria (DIC) (80). Type I does not smooth through space or time and force spatial and temporal neighbors to behave similarly; which we also assumed could potentially bias the Bayesian Network predictions.

### Data setup for training, testing, and validation

Since this is a hybridized technique, there were two ways the collected data were organized for modeling. The cases and expected cases were included in the Bayesian hierarchical spatial-temporal framework specified in section Relative risk estimation. The fitted responses from this model were used as observed values of relative risk for area *i* at time *t*. We did not include the vulnerability and resiliency indices or temperature and precipitation as covariates at this stage of the model.

#### Discretization

Bayesian networks often require discretization of continuous variables, but can accommodate continuous data and a mixture of discrete and continuous variables. However, many implementations of BN processes in machine learning require discrete data. Discretization also has numerous benefits for modeling and has the potential to aid model accuracy and computational complexity in some instances (81). We elected to discretize the model variables for this study to lower complexity and aid computational time. For relative risk and SMR discretization we used the typical 3 category risk model of low, medium and high and separated each class into two distinct levels for a total of 6 classes: 0–0.5, Low Low; 0.5–1.00 High Low; 1.0–1.25, Low Moderate; 1.25–1.50, High Moderate; 1.50–1.75, Low High; 1.75 and up, High High (the sensitivity analysis used an additional three classification schemes that are discussed in section Testing, training sets, validation, and sensitivity analysis). Since the CDC SVI values are approximately uniformly distributed they were discretized into four different groupings as follows: 0–0.25, Low; 0.25–0.50, Moderate; 0.50–0.75, High; 0.75–1.00, Extreme. The SoVI, NRI, and BRiC were discretized using a *k*-means clustering technique with four clusters to match the categories for the CDC SVI. The environmental variables were discretized with *k*-means with 15 different groupings across the entire time-frame. Fifteen classes for temperature was the lowest number of clusters possible that allowed each month to have more than one value. For example, with 14 classes the month of June 2020 had only one class which is an issue for the DBN inference engine. These variables are all approximately Gaussian distributed with positive skewness for temperature observed and predicted and negative skewness for precipitation observed and predicted. *k*-means clustering has been shown to be a reliable and robust method of discretization in supervised and unsupervised learning applications including Bayesian networks (82, 83).

#### Testing, training sets, validation, and sensitivity analysis

Data was separated into several training and testing sets. The initial training set included all data collected from March 1, 2020 to September 30, 2021. The complementary testing set included data for October 2021. We created three additional training sets (1) March 1, 2020 through February, 28 2021. (2) March 1, 2020 through November 30, 2020. (3) March 1, 2020 through August 31, 2020. Complementary testing sets for each training set consisted of data for the month immediately following the last month in the training set. These additional training and testing sets were used to further evaluate the DBN at three randomly selected discrete time intervals throughout the study period (September and December 2020, March 2021). Arranging the data in this way effectively simulates not having data for the month for which the forecast is made.

Another training set was created using a balanced dataset with *n* = 1,000 samples for all six classes. After training each model we tested the accuracy of the models trained with both approaches. We used cross-validation to compare alternative models to the DBN outputs and to compare alternative methods amongst themselves. Accuracy and Cohen's Kappa (*K*) were used for these comparisons (84). Models trained with all data available for prior months proved to be the more accurate (overall accuracy and Cohen's Kappa) training scenario (compared to the balanced training set) *via* these cross-validation parameters. DBN networks were also compared using cross-validation (total accuracy, Cohen's *K*), Akaike Information Criteria (AIC), Bayesian Information Criteria (BIC), and total number of free parameters (degrees of freedom) (84–86).

In order to test the sensitivity of the final DBN model to changes in classification scheme we tested three additional discretization categorizations, of relative risk and SMR, apart from the six-class model in section Discretization. These included: A nine class output with increments of 0.33 from zero to >2.66. A 12 class scheme with increments of 0.25 from zero to >2.75, and finally 16 classes with increments of 0.10 from zero to >3.00. These four classification schemes are evaluated with total accuracy and Cohen's *K* and should be sufficient to test the tendency of the model to correctly identify varying risk categories.

### Alternative modeling

We selected five alternative techniques that are commonly used in predictive analytics. The response from these five methods are used to compare to the outputs from the DBN.

#### Bayesian hierarchical spatial-temporal forecast

To compare a Bayesian hierarchical spatial-temporal forecast with our developed DBN we created a model which predicts the relative risk for October 2021 based on exchangeability with the link function to the likelihood (47, 87). This prediction $Y_{it}^{Pred}$, is based on information gathered by previously known values of *Y*_*it*_ (from prior months) based on


$$\begin{array}{l} p\left(Y_{it}^{Pred}|Y_{it}\right)=\frac{p\left(Y_{it},Y_{it}^{Pred}\right)}{p\left(Y_{it}\right)}\\ \text{from the conditional probability};\\ =\;\frac{\int p\left(Y_{it}^{Pred}|\;\xi \right)\;p\left(Y_{it}|\xi \right)p\left(\xi \right)d\xi }{p\left(Y_{it}\right)}\\ \text{by the property of exchangeability};\\ =\;\frac{\int p\left(Y_{it}^{Pred}|\xi \right)p\left(\xi |Y_{it}\right)p\left(Y_{it}\right)d\xi }{p\left(Y_{it}\right)}\\ \text{by application of Baye}{\text{s}}^{\prime }\text{ theorem};\\ =\;\int p\left(Y_{it}^{Pred}|\xi \right)p\left(\xi |Y_{it}\right)d\xi \end{array}$$

In this case ξ is a vector of the model parameters established with the suitable prior, p(ξ). We assume in this case that the variables being predicted are similar to previous observed cases and that $Y_{it}^{Pred}\;\&\;Y_{it}$ are generated by the same random processes generalized by the model parameters ξ. We did include the vulnerability and resiliency indices and observed/predicted temperature and observed/predicted precipitation as covariates in this model to aid the forecast. After prediction, these relative risk values were discretized using the same procedures outlined in the previous section and compared to the DBN model projections. This model is setup similarly to the model used to fit the observations for testing except that we do include the vulnerability and environmental measurements and do not include case numbers for October 2021 for which the forecast is made. This alternative method was developed in the R-INLA package (48).

#### Multinomial logistic regression

We also developed a multinomial logistic regression model to compare with the Bayesian network forecasts. Multinomial logistic regression (MNLR) behaves similarly to binary logistic regression except more than two categories can exist in the response. MNLR has been used in a number of health related studies where risk factors are linked to categorical outcomes (41, 88–92). In our MNLR setup we use K (6 categories) possible outcomes by running 5 independent binary logistic regression models:


$$\begin{array}{l} \;\ln\frac{\mathrm{Pr}(Y_{i}=1)}{\mathrm{Pr}(Y_{i}=K)}=\;\beta_{1}\cdot \;X_{i}\\ \;\ln\frac{\mathrm{Pr}(Y_{i}=2)}{\mathrm{Pr}(Y_{i}=K)}=\;\beta_{2}\cdot \;X_{i}\\ \;\cdots \cdots \\ \ln\frac{\mathrm{Pr}(Y_{i}=K-1)}{\mathrm{Pr}(Y_{i}=K)}=\;\beta_{K-1}\cdot \;X_{i} \end{array}$$

Summing the exponentiated solution for the probabilities of each class and knowing that all class probabilities sum to one:


$$\begin{array}{l} \mathrm{Pr}\left(Y_{i}=K\right)=1-\sum_{k=1}^{K-1}\mathrm{Pr}\left(Y_{i}=k\right)=1\\ \;-\;\sum_{k=1}^{K-1}\mathrm{Pr}\left(Y_{i}=K\right)e^{\beta_{k}\cdot X_{i}}\to \mathrm{Pr}\left(Y_{i}=K\right)\\ \;=\;\frac{1}{1+\;\sum_{k\;=\;1}^{K-1}e^{\beta_{k\cdot X_{i}}}} \end{array}$$

Then solving for each class probability.


$$\begin{array}{l} \;\mathrm{Pr}\left(Y_{i}=1\right)\frac{e^{\beta_{1}\cdot X_{i}}}{1+\;\sum_{k\;=\;1}^{K-1}e^{\beta_{k\cdot X_{i}}}}\\ \;\cdots \cdots \\ \mathrm{Pr}\left(Y_{i}=K-\;1\right)\frac{e^{\beta_{K-1}\cdot X_{i}}}{1+\;\sum_{k\;=\;1}^{K-1}e^{\beta_{k\cdot X_{i}}}} \end{array}$$

The MNLR model for this study was developed using the caret and nnet packages in R (93, 94).

#### Random forest regression

Random Forest Regression was also chosen as a technique to compare with the alternative methods and the Bayesian Network (95). Random Forests (RF) work by constructing numerous decision trees in the training step and averaging the prediction of individual trees within the forest. One key benefit of RF over other methods is they resist overfitting to the training set (96). RF has been used effectively in a number of epidemiological studies and they often outperform other techniques (97–103). Specifically, related to COVID-19 Muhammad et al. found a decision-tree model had the highest accuracy at predicting positive and negative cases of the disease (104). We implemented a Random Forest with Gini Impurity and calculated variance reduction with mean square error. Each decision tree in the forest is defined by:


$$\begin{array}{l} ni_{j}=w_{j}C_{j}-w_{left\left(j\right)}C_{left\left(j\right)}-w_{right\left(j\right)}C_{right\left(j\right)} \end{array}$$

Where *ni*_*j*_ is the importance of node j, *w*_*j*_ weighted number of samples for node j, *C*_*j*_ is the impurity at node j, *w*_*left*(*j*)_ is the child node on the left split on node j, *w*_*right*(*j*)_ is the child node on the right split from node j. The importance of each feature is calculated by:


$$fi_{i}=\frac{\sum_{j:\to i}ni_{j}}{\sum_{k\in all\;nodes}ni_{k}}$$

Each tree is normalized by:


$$normfi_{i}=\frac{fi_{i}}{\sum_{j\in all\;features}fi_{j}}$$

Then for the entire forest, feature importance is assigned by:


$$RFfi_{i}=\frac{\sum_{j\in all\;trees}normfi_{ij}}{T}$$

The RF model for this study was developed using the caret and randomForest packages in the R Statistical platform (93, 105).

#### Support vector machine

**Figure 3:**
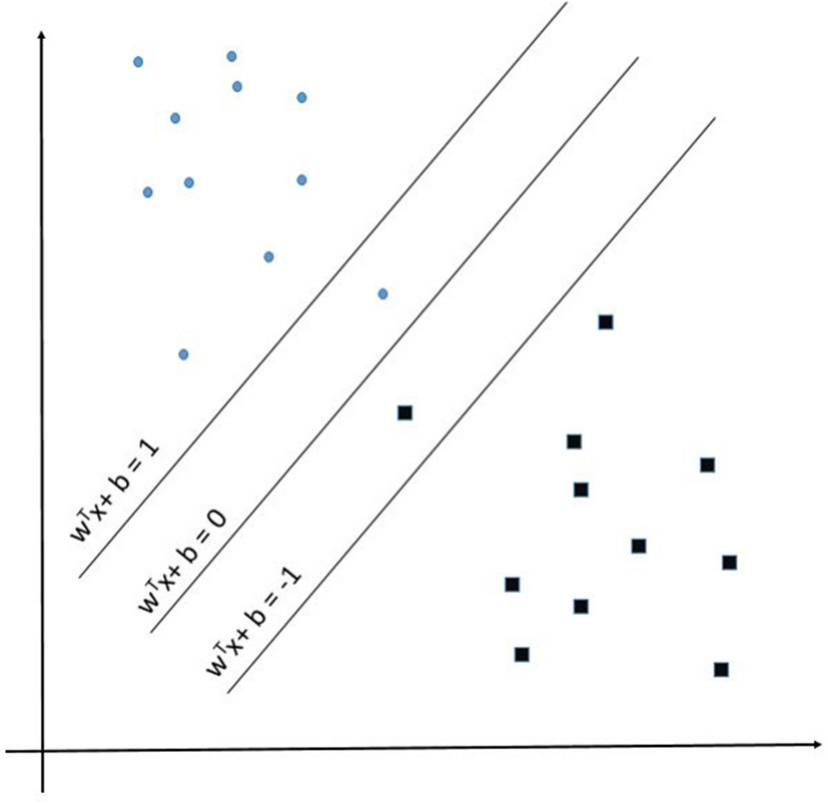
Support Vector Machine example representation showing the maximum-margin hyperplane W^T^x + b = 0.

Support Vector Machine (SVM) learning algorithms are widely considered one of the more robust prediction methods available (106–109). Muhammad et al. found SVM to have the highest sensitivity −93.34%—of the numerous examined machine learning techniques at predicting positive and negative cases of COVID-19 in Mexico (104). Given an input space with *p* dimensions the SVM learns a *p*-1 dimensional maximum-margin hyperplane (w^T^x – b = 0) separating the input data relative to the classes (1 and −1 in this example) defined in supervised learning (see Figure 3). Given a more complex *p*-dimensional non-linear classification the SVM uses the kernel trick which avoids the numerous issues with forcing linear learning functions to learn non-linear relationships. For our developed SVM model we used a radial basis function kernel defined by:


$$K(x,\overset{´}{x})=\exp\left(-\frac{{\left\|x-\;\overset{´}{x}\right\|}^{{}^{2}}}{2\sigma^{2}}\right)$$

$||x-\;\overset{´}{x}|{|}^{2}$ is the squared Euclidian distance between feature vectors *x* and $\overset{´}{x}$ and σ is the free parameter and is assigned a value of 0.1 in our model. Our alternative SVM model was developed using the caret and e1071 packages in the R Statistical platform (93, 110).

### Multinomial naïve Bayes

Naïve Bayes relies on Bayes theorem to calculate the probability of an event occurring relative to prior knowledge related to the event. In many ways it is similar to the Bayesian Network description and it is the simplest form of one. Multinomial naïve Bayes creates a feature vector histogram x_i_ with the number of times event *i* is observed. The likelihood of observation is:


$$p\left(x|C_{k}\right)=\;\frac{\left(\sum_{i=1}^{n}x_{i}\right)!}{\prod_{i=1}^{n}x_{i}!}\prod_{i=1}^{n}p_{ki}{}^{x_{i}}$$

Then through log-space reduction it becomes a linear classifier:


$$\begin{array}{l} \log p\left(C_{k}|x\right)\;\propto log\left(p\left(C_{k}\right)\overset{n}{\underset{i=1}{\prod }}p_{ki^{x_{i}}}\right)\\ =\log p\left(C_{k}\right)+\;\overset{n}{\underset{i=1}{\sum }}x_{i}\;\cdot logp_{ki}\\ =b+\;W_{k}^{T}X\\ b=\log p\left(C_{k}\right)\;\&\;w_{ki}=\log p_{ki}\\ C_{k}=Class\;\text{k}. \end{array}$$

Laplace smoothing was used to prevent the possibility of having a probability of zero in the conditional calculations. Multinomial naïve Bayes classification has been shown to be highly effective in a number of studies, especially as it relates to natural language processing, but also for discrete classes of observations (111–114). We used the caret and e1071 packages for R to develop the multinomial naïve Bayes model (93, 110).

## Results

**Figure 4:**
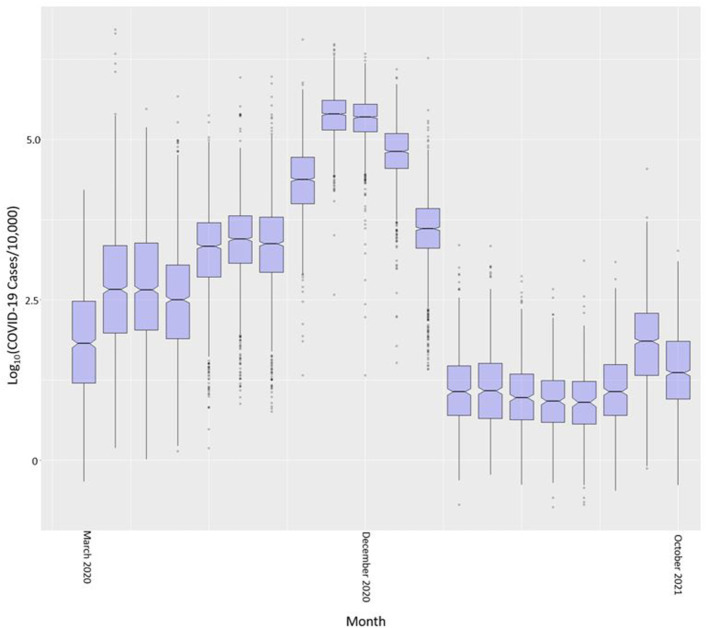
COVID-19 case rate per 10,000 (in common logarithm) by month across all Indiana census tracts.

The common logarithm of cases per census tract is shown in boxplots by month in Figure 4. We used the common logarithm because scaling the rates in this way allowed for a better visualization of the variable. COVID-19 cases in Indiana steadily increased from March 2020 through November and December of 2020 and significantly dropped after February of 2021. This drop coincides with the large scale vaccination efforts across the state. Cases began to rise again in September of 2021 with a drop into October 2021.

### Dynamic Bayesian Network specification

Multiple approaches can be used to learn the structure of a BN or DBN and specifying an appropriate structure is very important. There are several machine-learning techniques that will “learn” the structure of the network from available data. Examples include hill-climbing, gradient descent, incremental association Markov blanket (IAMB), naïve Bayes, and Tree-Augmented naïve Bayes (69, 115–118). An alternative is to construct the network's structure from expert advice and follow a pattern of causation; whether causation is true or not (119). In this study we specified the structure of our network with expert opinion following dependency of the variables from other studies.

In order to further evaluate the relevance of the selected variables, mean values of which are shown in Table 1, we added each into a preliminary DBN structure that included each variable along with the relative risk estimates as a first order Markov process. The output is presented in Table 2. The addition of each variable on its own did increase the accuracy and significantly lowered Akaike Information Criteria (AIC) and Bayesian Information Criteria (BIC). Including temperature and precipitation (each contemporaneous and first order Markov processes), and relative risk as a first and second order Markov process had the greatest effect on predictive accuracy, *K*, AIC, and BIC.

**Table 1:** Mean values by Indiana census tract for variables used in the DBN.

**Month–year**	**Mean posterior relative risk**	**Temp_real_**	**Precip_real_**	**Temp_predict_**	**Precip_predict_**	**CDC SVI**	**SoVI**	**NRI**	**BRiC**
March-20	0.979	7.789	103.888						
April-20	0.976	9.672	75.995						
May-20	1.015	15.834	130.735						
June-20	0.979	22.578	89.755						
July-20	1.004	25.856	103.786						
August-20	1.005	23.099	79.152						
September-20	0.395	19.455	36.429	21.276	82.767				
October-20	0.383	12.519	109.224						
November-20	1.029	8.057	73.983						
December-20	1.617	1.161	43.625	0.526	77.847	0.485	−0.0115	23.41	55.813
January-21	3.117	−0.925	58.443						
February-21	1.484	−4.513	50.767						
March-21	1.002	7.571	85.574	8.456	83.064				
April-21	0.999	11.535	70.775						
May-21	0.999	16.057	90.196						
June-21	0.999	23.566	149.785						
July-21	1.005	23.941	119.334						
August-21	1.041	24.736	83.189						
September-21	1.039	20.476	95.361						
October-21	1.035	16.218	168.309	15.897	75.565				

**Table 2:** Variables added to DBN independently.

	**Accuracy**	**95% CI**	**Kappa**	**AIC**	**BIC**
**RR 1st order**					
Markov process	0.3659	0.3415–0.3909	0.051	−116594.50	−118130.60
US CDC SVI	0.3779	0.3533–0.403	0.07	−117083.30	−121529.50
SoVI	0.3773	0.3487–0.3983	0.052	−117093.00	−121539.20
NRI	0.3759	0.3514–0.401	0.073	−117113.50	−121559.60
BRiC	0.3806	0.3559–0.4057	0.07	−117016.00	−121462.20
Temperature	0.3965	0.3717–0.4218	0.101	−119096.10	−129854.10
Precipitation	0.4824	0.4568–0.509	0.271	−152209.60	−269590.90
**RR 2nd order**					
Markov process	0.4032	0.3783–0.4285	0.165	−116035.80	−121956.90

Null information rate = 0.3606.

**Figure 5:**
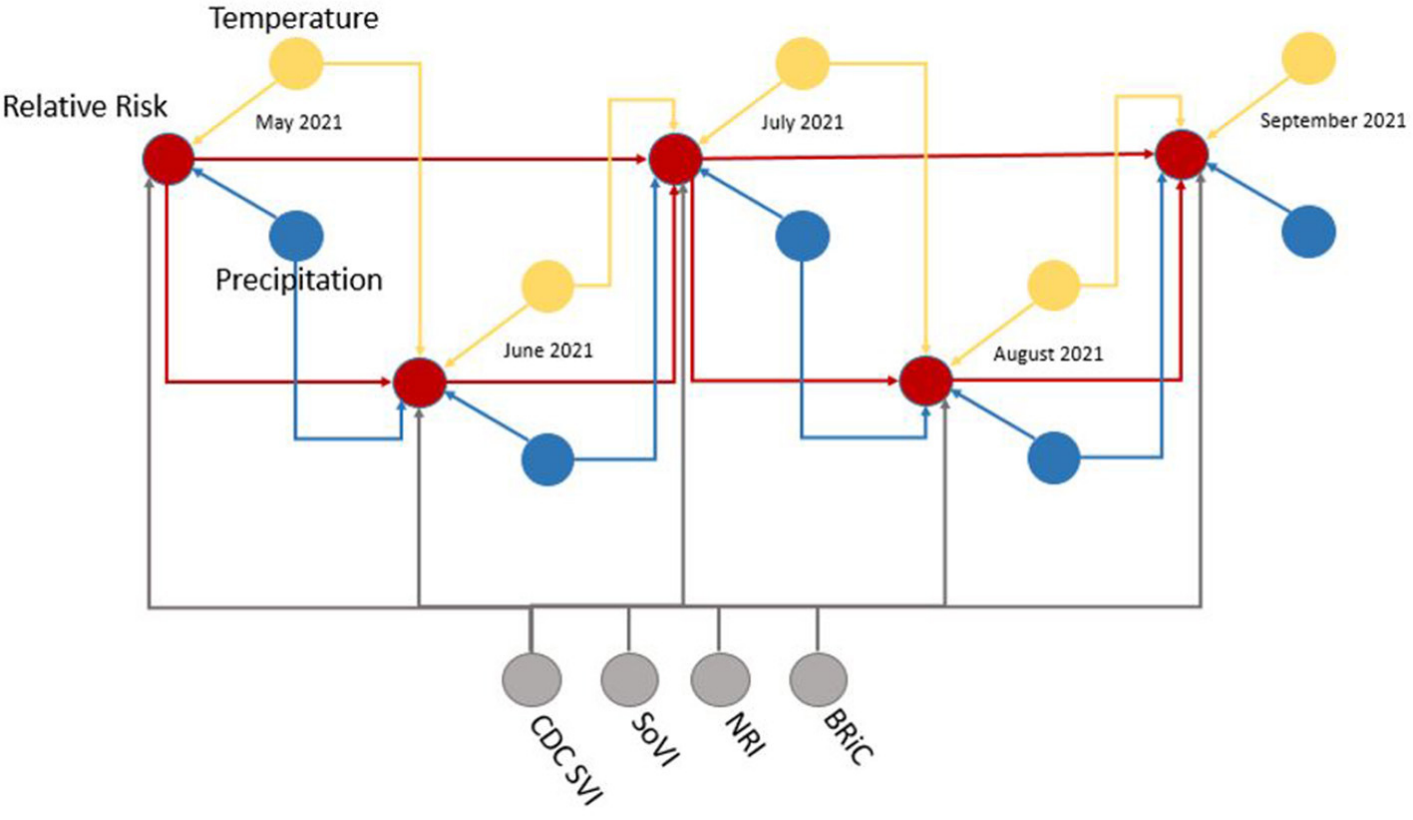
Example subset of recurrent structure of the DBN; yellow, temperature nodes; blue, precipitation nodes; gray, social vulnerability/resiliency index/NRI nodes; red, contemporaneous target, relative risk. For simplicity this DAG makes no distinction between predicted temperature from NEX-DCP30 and observed temperature from NLDAS. As the modeling moves forward in time, the NEX-DCP30 values are replaced by the NLDAS observations once the realization is available.

**Figure 6:**
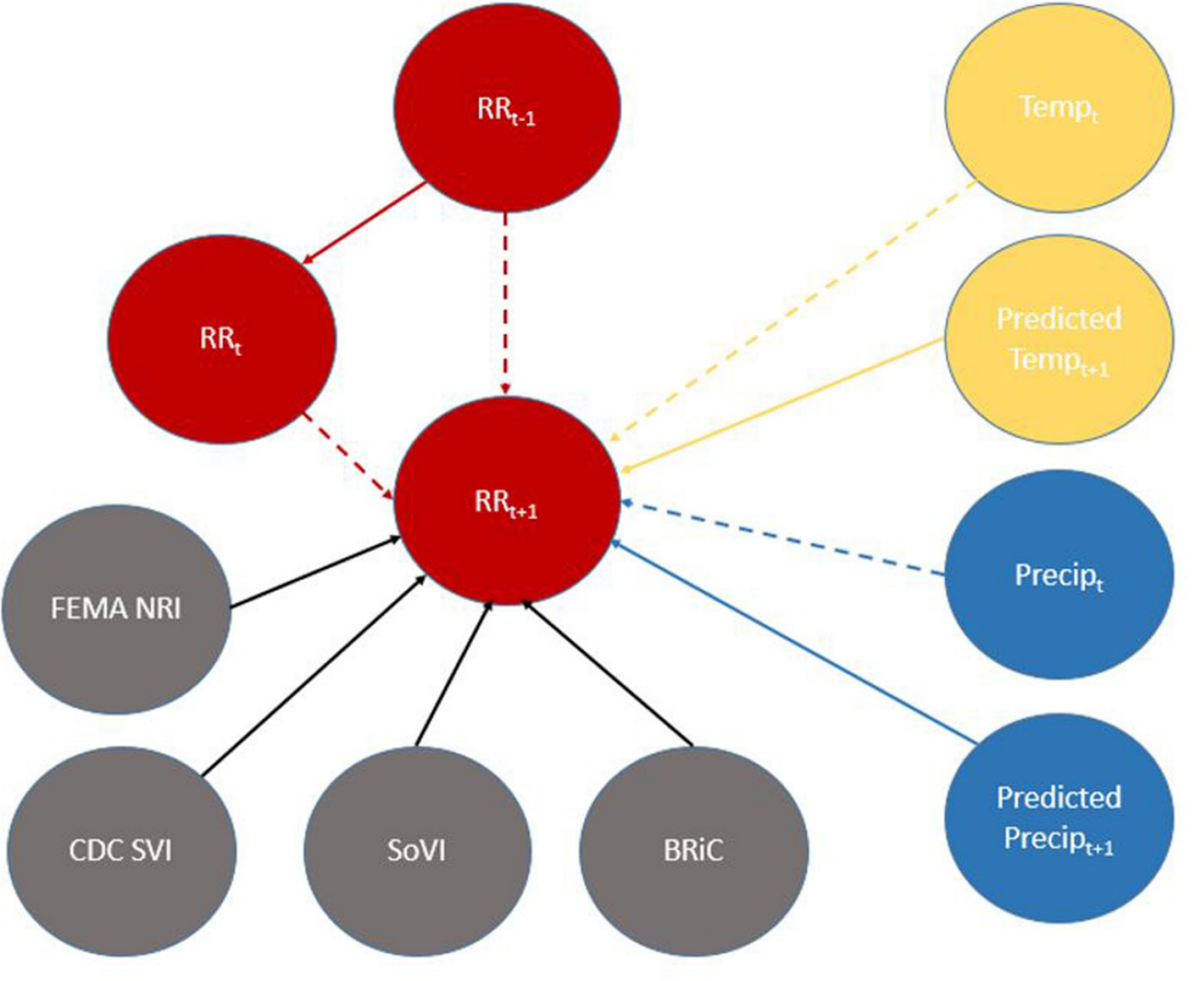
Directed Acyclic Graph (DAG) for (time-slice t) from DBN for predicting relative risk of COVID-19 infection. Dashed lines represent data input from a non-contemporaneous node/inter-time-slice (t-1).

To determine the overall network structure (DAG) as specified in Figures 5, 6, the selected variables were incrementally added beginning with the relative risk estimates of the previous months as a first order Markov process. At this step, variables (nodes,) were incrementally added and if the inclusion continued to improve the classification accuracy or lowered the AIC and BIC by at least 10 units the variable was retained (102, 120, 121). Through this process all selected variables improved the classification accuracy and lowered AIC and BIC significantly (see Table 3). In order to develop competing DBN models, we experimented with divorcing nodes from the target by adding the SoVI and BRIC values as direct inputs to NRI since its formulation contains those two metrics (DBN model #1). A second model (DBN model #2) used the temperature and precipitation of the previous month as an input to the contemporaneous temperature and precipitation nodes, rather than directly linked to relative risk (i.e., DBN model #1 and #3). Relative risk was also linked by its value from the previous month. The final DBN structure (DBN model #3), of which subsets are shown in Figures 5, 6, ultimately contained 64 nodes and had the most significant AIC and BIC values. In this architecture, the contemporaneous physical environment nodes were connected to values of the previous month. Social vulnerability nodes were also directly linked to the contemporaneous relative risk node. Finally, to further improve the model we introduced more memory into the relative risk estimation by making the relative risk linkages from past months into a second order Markov process where the predicted relative risk is dependent on the relative risk from the prior 2 months (122). This architecture ultimately contained 193 arcs connecting all the nodes with an average Markov Blanket of 16.41; as an example if this network were a fully connected DAG there would be $\left(\frac{n\left(n-1\right)}{2}\right)$ or 2016 arcs (9.57% of total possible). Each node has an average of 6.03 neighbors and the average branching is 3.02. Parameter learning was performed using maximum likelihood estimation in the bnlearn and dbnlearn packages (49, 123).

**Table 3:** Incremental addition of variables into the DBN.

	**Accuracy**	**95% CI**	**Kappa**	**AIC**	**BIC**
**RR 1st order**					
Markov process	0.37	0.34–0.39	0.05	−116594.50	−118130.60
US CDC SVI	0.38	0.35–0.40	0.07	−117083.30	−121529.50
SoVI	0.41	0.38–0.43	0.13	−119654.10	−135740.60
NRI	0.47	0.44–0.49	0.25	−132598.10	−195245.90
BRiC	0.61	0.58–0.63	0.46	−195858.90	−444751.90
Temperature	0.75	0.72–0.77	0.67	−1079762.20	−3688462.30
Precipitation	0.90	0.88–0.91	0.87	−128374752.50	−469307883.50
**RR 2nd order**					
Markov process	0.97	0.95–0.97	0.96	−659576242.50	−2375663790.20

Null information rate = 0.3606.

When viewed in its entirety this network matches prior research and conditional assumptions regarding COVID-19 infection and social and environmental metrics. For example, COVID-19 infection relative risk in July is conditionally dependent on average temperature in June and July and is conditionally independent of temperature measured in August. Similarly, July infection relative risk is conditionally dependent on the relative risk measured in May and June (2nd order Markov chain), but independent of that measured in August. In the case of our developed model, it may also be considered independent of that measured in April, however, since April is a part of the Markov chain of events it does have some bearing on a contemporaneous measurement; although clearly not as directly as those connected *via* arcs. This is an example of a process known as the “propagation of evidence” within the DBN structure which aids inference, classification and prediction.

### Model outputs

The results of fitting the DBN network and predicting relative risk categories for October 2021 are shown in Table 4. DBN model #3 (Figures 3, 4) has the highest accuracy and the least number of effective parameters of the examined DBN architectures. DBN model #2 is only slightly less accurate but with a significantly higher AIC and BIC. DBN model #2's architecture, differs from that in Figures 3, 4 as it links the prior month's temperature node to the current month's temperature node. This is the only difference between DBN #2 and DBN #3. DBN #1 is significantly less accurate with significantly higher AIC and BIC scores and a significant increase in effective parameters. The DBN #1 architecture is similar to DBN #2, except the BRIC and SoVI nodes are linked to the NRI node. This mimics the NRI score itself since its product includes the two variables (along with insurance loss, a variable which we did not include in this study).

**Table 4:** Diagnostic measurements of the developed predictive models.

**DBN model iteration**	**Nodes**	**Arcs**	**AIC**	**BIC**	**Effective parameters (DoF)**	**Accuracy**
DBN #1	64	156	−17248202.00	−62846642.00	3575	0.8902
DBN #2	64	194	−274603291.00	−1004163064.00	1764	0.9387
DBN #3	64	193	−792350979.00	−2897859858.00	1680	0.9534

Predictions for October 2021.

Table 5 presents the results of the alternative modeling techniques used to compare to the DBN forecasts. The SVM model with a radial basis kernel was found the be the most accurate with a 0.5853 accuracy rate and a *K* of 0.1651. The random forest prediction was very close to the SVM with a 0.5782 accuracy and a *K* of 0.1016. The naïve Bayes, MNLR and Bayesian hierarchical spatial-temporal projection did not produce accuracies that were statistically significant improvements over the null information rate of 0.5456 based on the 95% confidence intervals.

**Table 5:** Alternative model classification projections for October 2021 relative risk.

**Forecasting technique**	**Accuracy**	**Confidence interval**	**Cohen's *K***
Bayesian hierarchical S-T projection*	0.3442	0.3202–0.3689	0.0215
Multinomial logistic regression	0.5509	0.5253–0.5763	0.0075
Support vector machine (radial basis)	0.5853	0.5581–0.6086	0.1651
Random forest	0.5782	0.5527–0.6033	0.1016
Naïve Bayes	0.5552	0.4969–0.6124	0.1435

Null information rate = 0.5456.

^*^ = Discretized after relative risk prediction.

Tables 6–9 contain the confusion matrices for all 4 months where predictions were calculated with DBN #3. Prediction accuracy and confidence intervals were consistently above 95%. *K*, which measures observed accuracy relative to expected accuracy is consistently above 0.95. All sensitivity and specificity measures were above 0.900 with the majority of values exceeding 0.960. These observations reflect very good agreement between the predicted values and the realization/observed values in the study.

**Table 6:** Confusion matrix for October 2021 prediction of relative risk of COVID-19 infection.

	**Predicted**							
	***LL***	***HL***	***LM***	***HM***	***LH***	***HH***	***Sensitivity***	***Specificity***
**Realization**								
Relative risk 0.00–0.50 (LL)	11	1	0	0	0	0	1.000	0.999
Relative risk 0.50–1.00 (HL)	0	801	26	9	0	1	0.969	0.947
Relative risk 1.00–1.25 (LM)	0	18	503	5	0	0	0.946	0.976
Relative risk 1.25–1.50 (HM)	0	5	3	96	0	0	0.873	0.994
Relative risk 1.50–1.75 (LH)	0	0	0	0	17	0	1.000	1.000
Relative risk > 1.75 (HH)	0	2	0	0	0	5	0.833	0.998

Overall accuracy = 0.953.

95% Confidence Interval = 0.941–0.963.

No information rate = 0.361.

P < 2.2e-16.

Kappa = 0.918.

**Table 7:** Confusion matrix for March 2021 prediction of relative risk of COVID-19 infection.

	**Predicted**								
	***LL***	***HL***	***LM***	***HM***	***LH***	***HH***	***Sensitivity***	***Specificity***	
**Realization**									
Relative risk 0.00–0.50 (LL)	351	8	3	2	1	1	0.975	0.989	
Relative risk 0.50–1.00 (HL)	4	530	2	2	1	3	0.974	0.984	
Relative risk 1.00–1.25 (LM)	3	2	248	3	0	0	0.965	0.995	
Relative risk 1.25–1.50 (HM)	2	0	0	154	0	0	0.974	0.995	
Relative risk 1.50–1.75 (LH)	1	2	1	0	69	0	0.904	0.998	
Relative risk > 1.75 (HH)	0	3	1	1	0	105	0.955	0.996	

Overall accuracy = 0.968.

95% Confidence Interval = 0.958–0.976.

No information rate = 0.361.

P < 2.2e-16.

Kappa = 0.958.

**Table 8:** Confusion matrix for December 2020 prediction of relative risk of COVID-19 infection.

	**Predicted**							
	***LL***	***HL***	***LM***	***HM***	***LH***	***HH***	***Sensitivity***	***Specificity***
**Realization**								
Relative risk 0.00–0.50 (LL)	447	7	0	3	1	1	0.978	0.987
Relative risk 0.50–1.00 (HL)	7	545	2	2	2	0	0.973	0.986
Relative risk 1.00–1.25 (LM)	2	1	239	0	0	1	0.984	0.997
Relative risk 1.25–1.50 (HM)	0	2	1	89	1	2	0.916	0.995
Relative risk 1.50–1.75 (LH)	0	2	0	2	52	0	0.929	0.999
Relative risk > 1.75 (HH)	0	0	1	1	0	90	0.989	0.999

Overall Accuracy = 0.972.

95% Confidence Interval = 0.962–0.979.

No Information Rate = 0.372.

P < 2.2e-16.

Kappa = 0.962.

**Table 9:** Confusion matrix for September 2020 prediction of relative risk of COVID-19 infection.

	**Predicted**							
	***LL***	***HL***	***LM***	***HM***	***LH***	***HH***	***Sensitivity***	***Specificity***
**Realization**								
Relative risk 0.00–0.50 (LL)	338	2	1	1	0	0	0.983	0.997
Relative risk 0.50–1.00 (HL)	3	578	1	2	0	0	0.986	0.989
Relative risk 1.00–1.25 (LM)	2	2	264	1	0	1	0.974	0.997
Relative risk 1.25–1.50 (HM)	0	3	1	119	0	1	0.968	0.996
Relative risk 1.50–1.75 (LH)	0	0	1	0	68	0	0.986	0.999
Relative risk > 1.75 (HH)	0	0	2	1	0	111	0.991	0.999

Overall Accuracy = 0.982.

95% Confidence Interval = 0.974–0.988.

No Information Rate = 0.389.

P < 2.2e-16.

Kappa = 0.9761.

**Figure 7:**
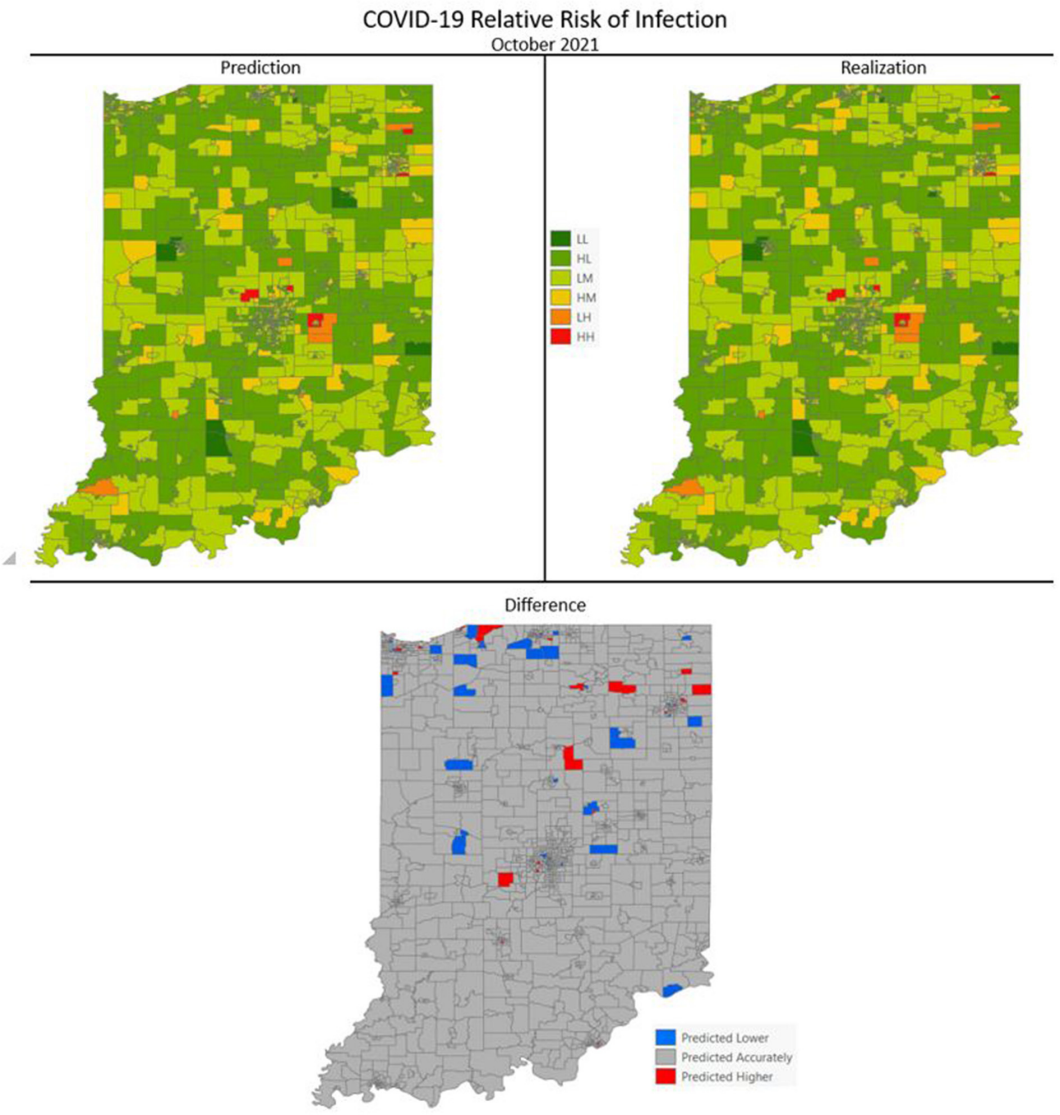
COVID-19 categorical relative risk of infection prediction and realization for October 2021.

**Figure 8:**
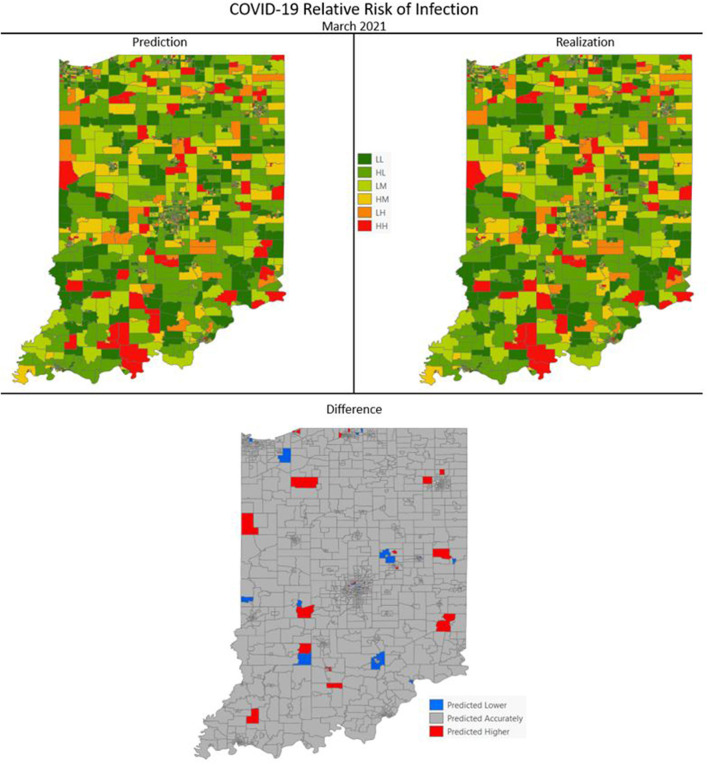
COVID-19 categorical relative risk of infection prediction and realization for March 2021.

**Figure 9:**
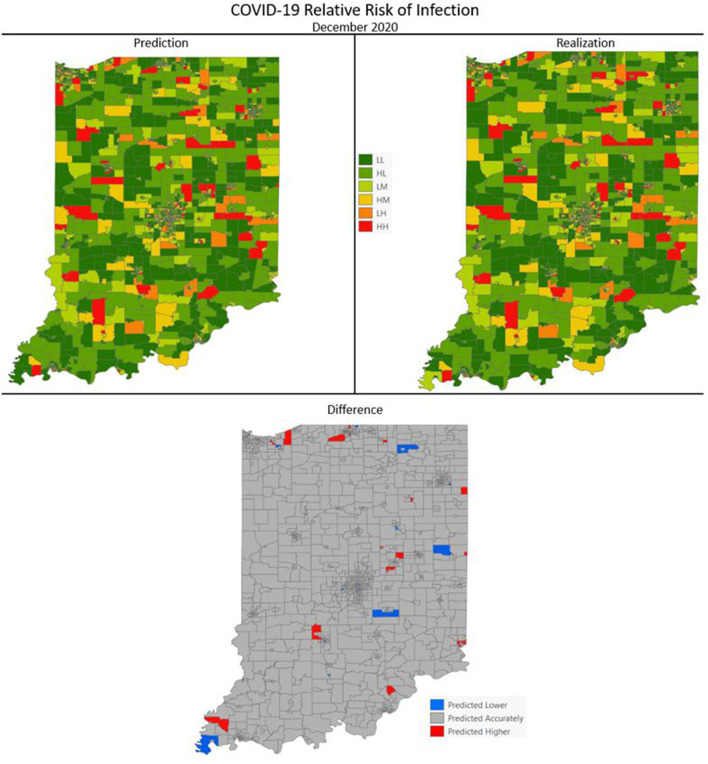
COVID-19 categorical relative risk infection prediction and realization for December 2020.

**Figure 10:**
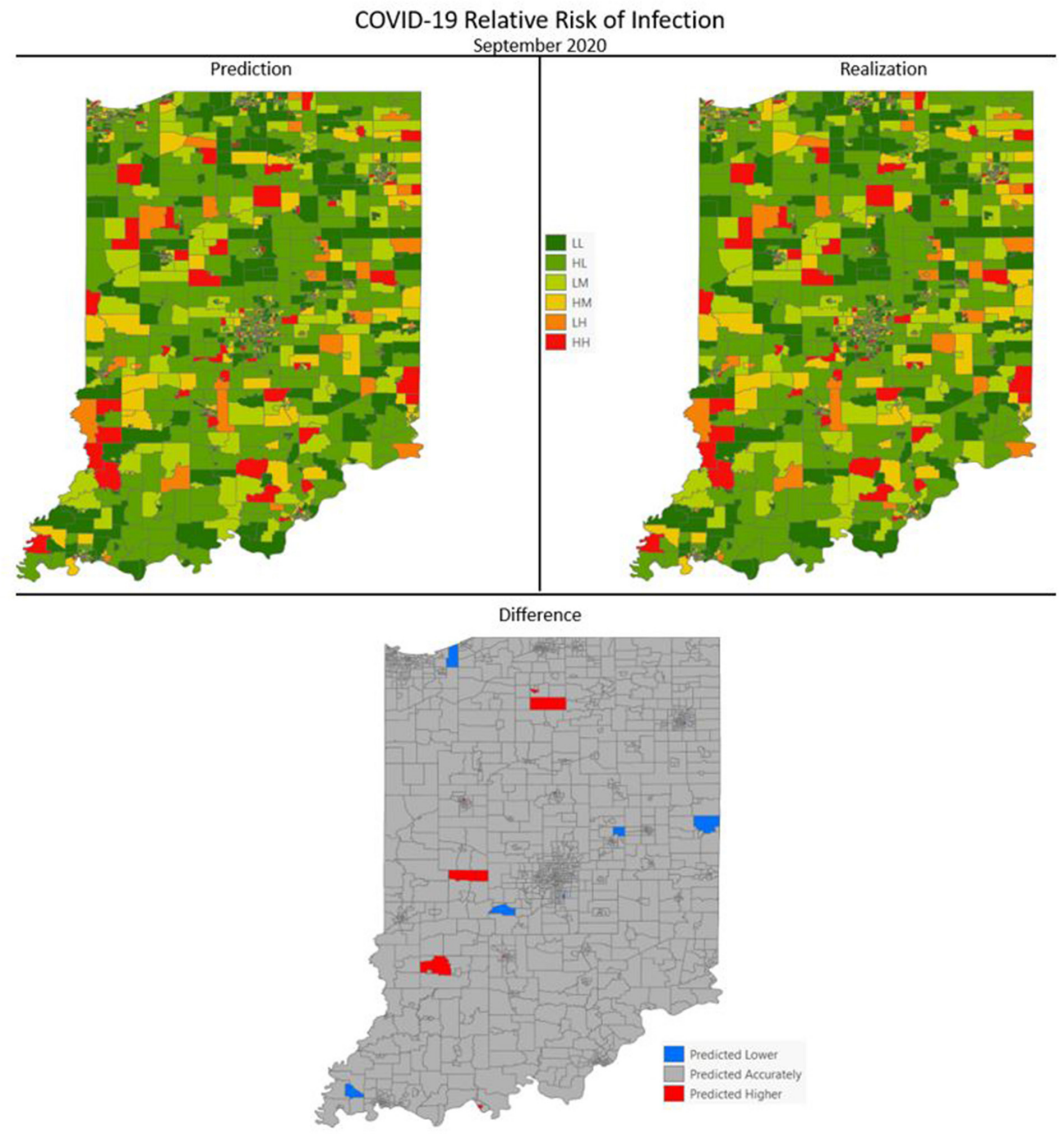
COVID-19 categorical relative risk of infection prediction and realization for September 2020.

Figures 7–10 are maps of the predicted categorical relative risk in the left-side panel, the realization on the right, and the difference below. The map showing difference is broken into 3 classes; under prediction, accurate prediction, and over prediction. The *p*-values for Moran's *i* were not statistically significant for the over and under predictions for each month; indicating a statistically significant probability of complete spatial randomness of the residuals from the DBN outputs.

Table 10 contains the metrics for the sensitivity analysis. We not only wanted to examine how different discretization patterns would impact the models but we wanted to investigate if using the (more easily calculated) SMR in place of modeled relative risk would also produce a viable result. For both relative risk and SMR overall accuracy and Cohen's K increase as the number of classes increase. The overall accuracy of the six-class model using relative risk was 0.953 and ranged to 0.979 for the 16-class scheme. The use of SMR, instead of modeled relative risk, did not seem to affect the end result and all models are statistically similar in their classification accuracy.

**Table 10:** Sensitivity analysis of the final DBN Model (DBN model #3).

**Modeled relative risk**	**Classes**			
	**6**	**9**	**12**	**16**
Accuracy	0.953	0.9627	0.9741	0.9789
95% Confidence Interval	0.941–0.963	0.952–0.972	0.965–0.982	0.971–0.984
No information rate	0.360	0.500	0.405	0.415
*P*-value	2.20E-16	2.20E-16	2.20E-16	2.20E-16
Kappa	0.918	0.943	0.965	0.969
**SMR**	**Classes**			
	**6**	**9**	**12**	**16**
Accuracy	0.958	0.9672	0.968	0.969
95% Confidence Interval	0.949–0.962	0.953–0.973	0.962–0.974	0.961–0.975
No information rate	0.379	0.473	0.332	0.426
*P*-value	2.20E-16	2.20E-16	2.20E-16	2.20E-16
Kappa	0.920	0.951	0.955	0.959

## Discussion

COVID-19 cases in Indiana steadily increased through the first month we made predictions, September 2020, a month which represented an inflection point as cases significantly increased through the remainder of the year. December 2020, another randomly selected month for DBN prediction, had the highest number of cases of all months in the study and follows the linear increase in cases from September. The third month in our predictive analysis, March 2021, succeeds another inflection point where cases dropped dramatically due to vaccine uptake in the population leading to an exponential decrease in confirmed cases. The final month, October 2021, shows a slight drop from those observed in September 2021. These characteristic trends within the data were initially thought to be potential problems for the DBN network and its predictive capabilities; especially with months following or being themselves inflection points. This proved to be less a challenge for the DBN predictions but it could explain some of the issues with the alternative modeling methods used.

The DBNs developed produce highly accurate predictions of categorical relative risk of COVID-19 infection at a very fine spatial scale (i.e., census tract). Notable from the incremental addition of nodes/variables to the network, all produced an increase in predictive accuracy and each lowered the AIC and BIC by > 488 units; well beyond the typical set threshold of 10 units (102, 120, 121). As we introduced variables into the DBN structure and even though there was overlap between the confidence intervals for accuracy (see Table 2) we retained the variables, relative risk of the previous month (a first order Markov process), the CDC SVI, and SoVI, because of the significant decrease in AIC and BIC. The improvements to the model, as measured by the information criteria, provides further evidence of association between measures of social vulnerability and COVID-19 infection rates supporting numerous studies (8, 11, 124–127). Our work also supports others finding network-based approaches to be superior in predicting COVID-19 risk (41, 42, 44).

Following the incremental introduction of variables into the network, using only the vulnerability/resiliency indices and the relative risk modeled for the prior month (1st order Markov process), we were able to achieve an accuracy of ~61%. Using only the vulnerability indices (CDC SVI and SoVI) and NRI produces a model with 47% accuracy, so the addition of the resiliency index, BRIC, adds 14% to the accuracy of the model; although none of these variables included individually offer superior performance compared to the null information rate (Table 2). The BRIC index is only available by county but does add significantly to the model. The inclusion of the dynamic environmental variables adds nearly 30% to the overall accuracy and significantly lowers AIC and BIC by orders of magnitude. The impact on predictive accuracy from average daily maximum temperature and precipitation (monthly sum) supports other studies finding correspondence between these two environmental measurements and COVID-19 cases of infection (11, 29–32). The order in which the variables were added to the model provides no remarkable change to the metrics shown in Table 3 and the overall predictive effect is not altered by a different order of introduction.

All three DBN models significantly outperformed the Bayesian hierarchical spatial-temporal forecast, MNLR, SVM, random forest and naïve Bayes estimations. The best alternative forecasting technique was the SVM model with a radial basis kernel, providing an accuracy of 0.5853 and a relatively low *K* (0.1651). The difference in performance between the alternative methods and the DBN estimates is remarkable. For the dataset under examination, the DBN with its approach of creating conditional and marginal probability tables based on the DAG modeling the data, has superior discriminatory power compared to the alternatives. This is likely due to the superior ability of the DBN framework to model non-linear and complex interactions between observations. Perhaps most surprising is how poorly naïve Bayes performed with a *K* of 0.1435 and an accuracy (0.5552) that was not statistically significant relative to the null information rate. As discussed previously, naïve Bayes is considered a very simplistic Bayesian network and in this case would be modeled as the predictive class being directly linked to each individual node (directionally, predictive class -> node); in this case only 63 arcs directed to the explanatory variables. Naïve Bayes is best used for unrelated classes of observations and our classes may be interrelated enough to negatively impact the prediction. The non-dynamic nature of the naïve Bayes model, however, is likely the greatest contributing factor to its insignificant prediction as it is not especially well-suited to time series observations.

The poor performance of the Bayesian hierarchical spatial-temporal projection is also notable. The only COVID-19 predictive study we found using this framework was a compartmental SEIR architecture, embedded in the hierarchy, used to estimate relative risk with a convolutional CAR model adjusting for structured spatial dependency and unstructured spatial heterogeneity (43). However, unlike Sartorius et al. (43), we did not include compartmental components in our modeling or factor in population mobility which make it less comparable. Our forecast using this approach is based on a similar model of the type used for fitting the COVID-19 cases to create observed relative risk estimates. This projection is based on exchangeability *via* a link function to the likelihood of the model for data from March 1, 2020 through September 30, 2021. This technique produced the poorest projection of relative risk estimates for October 2021 from all the models calculated and is perhaps due to only using the distribution of the likelihood. The similarity between the accuracy of this forecast and the separate predictions observed from placing the variables individually into the DBN structure (Table 2) adds some support to this notion.

Difference maps, between predicted categories and observed (the bottom figure in Figures 7–10), show the residuals to be randomly distributed in space with a very even distribution between over-predicted and under-predicted classes. Recall, the predictions utilize data from NASA NEX-DCP30 to forecast the average environmental conditions for the target month. However, once observed values are available (~1–2 days after the end of the month) and after a prediction is made with the DBN, we replace the environmental variables with the observed NLDAS values. This process mimics what would be needed in an actual predictive decision-making environment. Given a need to calculate relative risk predictions a month into the future, we collect values from NEX-DCP30 to obtain the forecast for the month and run the DBN prediction. As time progresses, the actual environmental variables are available and updated to observed values. Continuing this recursive process adds greater memory to the model, mimics the DBN structure and validates the predictions. This process can be automated in its entirety with scripting.

Reasons for misclassification of certain areas are difficult to ascertain. Residuals are randomly distributed in space and no census tract is over or under predicted for more than one time frame. In other words, no census tract in the study is misclassified more than once. Some of the reasons for misclassification could be the spatial resolution of datasets we use to calculate environmental conditions within a census tract. The spatial resolution of these environmental datasets are courser than a typical census tract, especially in urban areas, and could add uncertainty to the classification. However, we are achieving a very high classification accuracy with DBN model #3 and would not expect accuracy to be 100%.

Bayesian networks are incredibly flexible and have the added benefit of allowing one to relatively easily conduct “what-if” analyses. This is often performed in decision-making environments and is not as easily performed in deep learning and artificial neural networks and practically intractable in regression-based techniques (59, 119). For example, if social vulnerability were lowered for a specific area and resiliency were increased we could calculate what effect that would have on our predictions for the location. Also, one could determine where performing such an intervention would likely be most impactful. Furthermore, the network is extensible, additional nodes could be added to the network such as one representing community vaccination efforts. This could simply be a Boolean node or something with a more complex state structure but a DBN is flexible enough to allow for its inclusion in future iterations.

This research, apart from adding to prior studies seeking to forecast infection at a fine spatial scale such as a census tract or the U.K. equivalent, mid-layer super output area (MSOA), suggests network-based approaches are superior to alternative methods. Much of the work dealing with larger scale forecasts utilizes artificial neural networks (ANN), with differing depths of learning and multiple architectures (i.e., LSTM, CNN), time series analysis using autoregressive techniques, Bayesian hierarchical spatiotemporal methods, and regression trees. Due to their more limited number, there are less examples forecasting infection at smaller scales. The few that exist also tend to focus on network-based approaches and Bayesian hierarchical methods of dealing with spatial, temporal and spatiotemporal interactions (41–44). Our findings add support to these studies suggesting that network-based approaches and accounting for spatial and temporal structure, in a hierarchical manner, blend together well.

### Limitations

As with all modeling techniques there are caveats and limitations. The accuracy is likely to be affected if the class structure were altered. For example, choosing different boundaries for categorizing the relative risk estimates could yield differences in accuracy. Table 10 contains the overall classification accuracy and Cohen's K (Kappa) for three alternative relative risk categorizations; apart from the presented six-class model. The overall accuracy gained from separating the relative risk categories in this way is not statistically significant relative to the six-class model. Additionally, since relative risk is somewhat difficult to calculate we compared the same categorizations using SMR. Similarly, using the Bayesian-modeled relative risk does not provide a statistically significant improvement over SMR; which is much more easily computed. However, one strength of using Bayesian-modeled relative risk is the ability to account for spatial and temporal structure in the data. Due to these characteristics and its capabilities with sparse data, which is more common in disease mapping studies, we thought it prudent to highlight the effectiveness of the more sophisticated model; further emphasizing the extensibility of the DBN methodology. Additionally, risk is often stratified into three categories of Low, Moderate and High with little guidance on where boundaries are in representative data; which are often defined by the distribution. We proceeded with six categories of risk, believing it more illustrative of the spatial variations in relative risk than simply 3 categories and more easily interpretable than nine, 12 or 16 classes. From our stratification it is easy to see relative risk values that are greater than the study area average at the time. Any census tract that is in the Low Moderate category or above has a greater than average relative risk of infection.

We also did not evaluate the model's predictive capabilities beyond a month into the future. The predictive accuracy of the model is likely to suffer if predictions are made beyond 1 month. However, it would be relatively easy to implement such a temporal extension to the modeling. In a similar vein, accuracy is likely to be different if we decompose the data into weekly discrete time increments to make forecasts with finer temporal specificity. We settled on monthly predictions because many public health agencies lack the capacity to act on daily and/or weekly scenarios. Monthly discrete intervals seemed to likely be the best fit for decision-making activities.

The use of county-level and census tract-level data could be considered a limitation due to using zonal calculations on the environmental variables. The environmental variables have different spatial resolutions in their original format (NLDAS = 13,945 m and NEX-DCP30 = 927.67 m). Clearly, these pixels are larger than many census tracts, especially in urban areas. Utilizing a dataset with a more enhanced spatial resolution would likely alter the results. There are likely to be important sub-census tract-level variations that are missed by these large spatial resolution datasets.

There has been recent research emphasis placed on modeling the effect of under-reporting of COVID-19 and other infectious diseases (128–130). We did not account for this in our modeling due to some of the intrinsic limitations in INLA (the package in R used for the Bayesian hierarchical modeling); lacking the ability to directly account for this (47). However, it is likely that if this effect were accounted for, the DBN would capture a significant portion of its effect and produce comparably accurate models. Future efforts should look to account for under-reporting and/or misreporting, especially in infectious disease research.

A final consideration is the computational intensity of the developed models. The Bayesian hierarchical spatial-temporal modeling takes several hours to compute on a relatively robust workstation (as of 2021) and can take days if it is necessary to recalculate conditional predictive ordinate values. The computation time is significantly improved when performed in high-performance computing environments. Our final Bayesian hierarchical spatial-temporal model used for validation was developed in Indiana University's High Performance Computing environment using parallel processing (131). DBN #3 similarly is ~100X more computationally intensive than the alternative methods examined.

## Conclusions

We developed a hybridized approach to forecasting categorical relative risk estimates at the census tract-level on monthly timeframes. This approach used a Bayesian hierarchical spatial-temporal model to calculate observed relative risk and a DBN to make the predictions. The Bayesian hierarchical technique has the added benefit of accounting for spatial and serial autocorrelation and other random effects within the relative risk estimate. By using this output, spatial and temporal issues are accounted for removing the need to introduce nodes taking these random effects into account; adding to the complexity of the network. The three DBNs developed all outperformed the alternative methods we used for comparison. There may be additional alternative techniques that could outperform the DBN on this dataset (i.e., LSTM, RNN, CNN), but these would likely be more difficult to implement and would not allow straightforward decomposition of the network's parameters determining variable importance; something the conditional and marginal probability tables in the DBN easily permit. Furthermore, it is more difficult and in many cases impossible to include expert opinion on data relationships in the specification of these alternative methods.

Even though our hybridized approach generated highly accurate predictions more work is needed that fosters predictive decision support in public health applications. The current COVID-19 pandemic has illustrated the need for these types of models to communication risk to the public and to support targeted intervention activities such as prioritizing communities for vaccination. The month-long predictive timeframe from our model fits squarely in the decision-making window of most public health agencies and makes it ideal for “what-if” analyses. For example, by adding a Vaccination node to our model, we could model the effects in a community after a vaccination campaign and use such outputs for prioritizing locations. Furthermore, we can readily incorporate the impact social vulnerability and resiliency have on the detriment or betterment of a community.

Further research efforts are needed in using both Bayesian and Dynamic Bayesian Networks in epidemiological studies. Unfortunately, examples of DBN approaches to dynamic epidemiological processes are low in number relative to other techniques used in machine learning and AI. A significant benefit of using this technique is its allowance for the inclusion of expert opinion and offers highly accurate and robust methods of classification and projection. They are also extensible by allowing future nodes to be added taking into account further considerations priming them for “what-if” analyses. The research presented here showcases the capabilities of DBN models to interface with other techniques and to provide an accurate and robust forecast. DBN-type models should be among the core techniques in early warning and response to pandemics or local-scale epidemics.

## Data availability statement

The raw data supporting the conclusions of this article will be made available by the authors, through IUPUI DataWorks. http://dataworks.iupui.edu.

## Ethics statement

The studies involving human participants were reviewed and approved by Indiana University Institutional Review Board. Written informed consent from the participants' legal guardian/next of kin was not required to participate in this study in accordance with the national legislation and the institutional requirements.

## Author contributions

DJ designed the study, collected data, conducted analysis, and wrote the majority of the manuscript. VL assisted in analysis and design and contributed to methods, results, discussion, and conclusion portions of the manuscript. All authors contributed to the article and approved the submitted version.

## Funding

This research was funded by the Office of the Vice President for Research at Indiana University and the Office of the Vice Chancellor for Research at Indiana University—Purdue University at Indianapolis.

## Conflict of interest

The authors declare that the research was conducted in the absence of any commercial or financial relationships that could be construed as a potential conflict of interest.

## Publisher's note

All claims expressed in this article are solely those of the authors and do not necessarily represent those of their affiliated organizations, or those of the publisher, the editors and the reviewers. Any product that may be evaluated in this article, or claim that may be made by its manufacturer, is not guaranteed or endorsed by the publisher.

## References

[1] Li J Lai S Gao GF Shi W. The emergence, genomic diversity and global spread of SARS-CoV-2. Nature. (2021) 600:408–18. 10.1038/s41586-021-04188-6 34880490

[2] Ritchie H Mathieu E Rodés-Guirao L Appel C Giattino C Ortiz-Ospina E. Coronavirus Pandemic (COVID-19). Our World in Data. (2020). Available online at: https://ourworldindata.org/covid-deaths

[3] Marani M Katul GG Pan WK Parolari AJ. Intensity and frequency of extreme novel epidemics. PNAS. (2021) 118:e2105482118. 10.1073/pnas.2105482118 34426498 PMC8536331

[4] Bansal A Padappayil RP Garg C Singal A Gupta M Klein A. Utility of Artificial Intelligence amidst the COVID 19 pandemic: a review. J Med Syst. (2020) 44:156. 10.1007/s10916-020-01617-3 32740678 PMC7395799

[5] Jayatilaka G Hassan J Marikkar U Perera R Sritharan S Weligampola H. Use of Artificial Intelligence on spatio-temporal data to generate insights during COVID-19 pandemic: a review. (2020) 2020.11.22.20232959. 10.1101/2020.11.22.20232959

[6] Mollalo A Rivera KM Vahedi B. Artificial neural network modeling of novel coronavirus (COVID-19) incidence rates across the continental United States. Int J Environ Res Public Health. (2020) 17:4204. 10.3390/ijerph17124204 32545581 PMC7344609

[7] Alcendor DJ. Racial disparities-associated COVID-19 mortality among minority populations in the US. J Clin Med. (2020) 9:2442. 10.3390/jcm9082442 32751633 PMC7466083

[8] Jackson SL Derakhshan S Blackwood L Lee L Huang Q Habets M. Spatial disparities of COVID-19 cases and fatalities in United States counties. Int J Environ Res Public Health. (2021) 18:8259. 10.3390/ijerph18168259 34444007 PMC8394063

[9] Tan SB deSouza P Raifman M. Structural racism and COVID-19 in the USA: a county-level empirical analysis. J Racial Ethnic Health Disparities. (2021) 9:236–46. 10.1007/s40615-020-00948-8 33469868 PMC7815192

[10] Finch WH Hernández Finch ME. Poverty and COVID-19: rates of incidence and deaths in the United States during the first 10 weeks of the pandemic. Front Sociol. (2020). 10.3389/fsoc.2020.00047 33869454 PMC8022686

[11] Johnson DP Ravi N Braneon CV. Spatiotemporal associations between social vulnerablity, environmental measurements, and COVID-19 in the conterminous United States. GeoHealth. (2021) 5:8. 10.1002/essoar.10506630.2 34377879 PMC8335698

[12] Patel JA Nielsen FBH Badiani AA Assi S Unadkat VA Patel B. Poverty, inequality and COVID-19: the forgotten vulnerable. Public Health. (2020) 183:110–1. 10.1016/j.puhe.2020.05.006 32502699 PMC7221360

[13] CDC's Social Vulnerability Index (SVI) (2021). Available online at: https://www.atsdr.cdc.gov/placeandhealth/svi/index.html

[14] Flanagan BE Gregory EW Hallisey EJ Heitgerd JL Lewis B. A social vulnerability index for disaster management. J Homeland Security Emergency Manage. (2011) 8:24. 10.2202/1547-7355.1792

[15] Cutter SL Boruff BJ Shirley WL. Social vulnerability to environmental hazards^*^. Soc Sci Q. (2003) 84:242–61. 10.1111/1540-6237.8402002

[16] Kaiser HF. The application of electronic computers to factor analysis. Educ Psychol Measure. (1960) 20:141–51. 10.1177/001316446002000116

[17] VMAP (2021). Available online at: https://www.vulnerabilitymap.org/Mapping-Tools/Social-Vulnerability

[18] Wilson B. bradleyswilson/soviR (2020). Available online at: https://github.com/bradleyswilson/soviR

[19] Xiong H. SoVI (2019). Available online at: https://github.com/hy-xiong/SoVI

[20] Cutter S Burton C Emrich C. Disaster resilience indicators for benchmarking baseline conditions. J Homeland Security Emerg Manage. (2010) 7:1. 10.2202/1547-7355.1732

[21] Cutter SL Barnes L Berry M Burton C Evans E Tate E. A place-based model for understanding community resilience to natural disasters. Global Environ Change. (2008) 18:598–606. 10.1016/j.gloenvcha.2008.07.013

[22] Chandra A Williams M Plough A Stayton A Wells KB Horta M. Getting actionable about community resilience: the Los Angeles county community disaster resilience project. Am J Public Health. (2013) 103:1181–9. 10.2105/AJPH.2013.301270 23678906 PMC3682620

[23] Gu J Chao J Chen W Xu H Zhang R He T. Multimorbidity and health-related quality of life among the community-dwelling elderly: a longitudinal study. Arch Gerontol Geriatr. (2018) 74:133–40. 10.1016/j.archger.2017.10.019 29096228

[24] Saravanan V Garren SJ. Baseline framework for assessing community resilience using a balanced index approach and spatial autocorrelation in the Mill river watershed, Nassau County, New York. Int J Disaster Risk Reduction. (2021) 66:102621. 10.1016/j.ijdrr.2021.102621

[25] Xu W Xiang L Proverbs D Xiong S. The influence of COVID-19 on community disaster resilience. Int J Environ Res Public Health. (2021) 18:88. 10.3390/ijerph18010088 33374318 PMC7795082

[26] Bashir MF Ma B Bilal Komal B Bashir MA Tan D. Correlation between climate indicators and COVID-19 pandemic in New York, USA. Sci Total Environ. (2020) 728:138835. 10.1016/j.scitotenv.2020.138835 32334162 PMC7195034

[27] Daanen H Bose-O'Reilly S Brearley M Flouris DA Gerrett NM Huynen M. COVID-19 and thermoregulation-related problems: practical recommendations. Temperature. (2021) 8:1–11. 10.1080/23328940.2020.1790971 33553500 PMC7849778

[28] Haque SE Rahman M. Association between temperature, humidity, and COVID-19 outbreaks in Bangladesh. Environ Sci Policy. (2020) 114:253–5. 10.1016/j.envsci.2020.08.012 32863760 PMC7447231

[29] Hassan MM Zowalaty MEE Khan SA Islam A Nayem MRK Järhult JD. Role of environmental temperature on the attack rate and case fatality rate of coronavirus disease 2019 (COVID-19) pandemic. Infection Ecol Epidemiol. (2020) 10:1792620. 10.1080/20008686.2020.1792620 32944163 PMC7480504

[30] Iqbal N Fareed Z Shahzad F He X Shahzad U Lina M. The nexus between COVID-19, temperature and exchange rate in Wuhan city: new findings from partial and multiple wavelet coherence. Sci Total Environ. (2020) 729:138916. 10.1016/j.scitotenv.2020.138916 32388129 PMC7194511

[31] Menebo MM. Temperature and precipitation associate with COVID-19 new daily cases: a correlation study between weather and COVID-19 pandemic in Oslo, Norway. Sci Total Environ. (2020) 737:139659. 10.1016/j.scitotenv.2020.139659 32492607 PMC7258804

[32] Prata DN Rodrigues W Bermejo PH. Temperature significantly changes COVID-19 transmission in (sub)tropical cities of Brazil. Sci Total Environ. (2020) 729:138862. 10.1016/j.scitotenv.2020.138862 32361443 PMC7182516

[33] Shi P Dong Y Yan H Zhao C Li X Liu W. Impact of temperature on the dynamics of the COVID-19 outbreak in China. Sci Total Environ. (2020) 728:138890. 10.1016/j.scitotenv.2020.138890 32339844 PMC7177086

[34] Li AY Hannah TC Durbin JR Dreher N McAuley FM Marayati NF. Multivariate analysis of black race and environmental temperature on COVID-19 in the US. Am J Med Sci. (2020) 360:348–56. 10.1016/j.amjms.2020.06.015 32709397 PMC7305735

[35] Barnett J Lambert S Fry I. The hazards of indicators: insights from the environmental vulnerability index. Ann Assoc Am Geograph. (2008) 98:102–19. 10.1080/0004560070173431532612364

[36] Ho HC Wong MS Man HY Shi Y Abbas S. Neighborhood-based subjective environmental vulnerability index for community health assessment: development, validation and evaluation. Sci Total Environ. (2019) 654:1082–90. 10.1016/j.scitotenv.2018.11.136 30841383

[37] Johnson DP Stanforth A Lulla V Luber G. Developing an applied extreme heat vulnerability index utilizing socioeconomic and environmental data. Appl Geogr. (2012) 35:23–31. 10.1016/j.apgeog.2012.04.006

[38] Kaly U Briguglio L McLeod H Schmall S Pratt C Pal R. Environmental Vulnerability Index (EVI) to summarise national environmental vulnerability profiles. SOPAC. (1999).

[39] Kaly U Pratt C Mitchell J. The Environmental Vulnerability Index 2004. Suva: South Pacific Applied Geoscience Commission (SOPAC) (2004).

[40] National Risk Index | FEMA.gov. (2021). Available online at: https://hazards.fema.gov/nri/

[41] Achterberg MA Prasse B Ma L Trajanovski S Kitsak M Van Mieghem P. Comparing the accuracy of several network-based COVID-19 prediction algorithms. Int J Forecast. (2020) 38:489–504. 10.1016/j.ijforecast.2020.10.001 33071402 PMC7546239

[42] Prasse B Achterberg MA Ma L Van Mieghem P. Network-inference-based prediction of the COVID-19 epidemic outbreak in the Chinese province Hubei. Appl Netw Sci. (2020) 5:35. 10.1007/s41109-020-00274-2 32835088 PMC7341469

[43] Sartorius B Lawson AB Pullan RL. Modelling and predicting the spatio-temporal spread of COVID-19, associated deaths and impact of key risk factors in England. Sci Rep. (2021) 11:5378. 10.1038/s41598-021-83780-2 33686125 PMC7940626

[44] Vitale V D'Urso P De Giovanni L. Spatio-temporal object-oriented Bayesian Network modelling of the COVID-19 Italian outbreak data. Spatial Statistics. (2021) 100529. 10.1016/j.spasta.2021.100529 34277332 PMC8277433

[45] Højsgaard S. Graphical independence networks with the gRain Package for R. J Statistical Softw. (2012) 46:1–26. 10.18637/jss.v046.i10

[46] Nagarajan R Scutari M Lèbre S. Bayesian networks in the presence of temporal information. In: Nagarajan R Scutari M Lèbre S, editors. Bayesian Networks in R: with Applications in Systems Biology. New York, NY: Springer (2013). p. 59–83. Available online at: 10.1007/978-1-4614-6446-4_3 10.1007/978-1-4614-6446-4_3

[47] R-INLA Project. Available online at: https://www.r-inla.org/

[48] Rue H Martino S Chopin N. Approximate Bayesian inference for latent Gaussian models by using integrated nested Laplace approximations. J R Statistical Soc Ser B. (2009) 71:319–92. 10.1111/j.1467-9868.2008.00700.x24416633

[49] Scutari M. bnlearn - Bayesian Network Structure Learning. (2021). Available online at: https://www.bnlearn.com/

[50] McDonald CJ Overhage JM Barnes M Schadow G Blevins L Dexter PR. The Indiana network for patient care: a working local health information infrastructure. Health Affairs. (2005) 24:1214–20. 10.1377/hlthaff.24.5.1214 16162565

[51] Regenstrief Institute. How We Bring the Data to You. Regenstrief Institute (2021). Available online at: https://www.regenstrief.org/rds/data/

[52] Aybar C cre Qiusheng W Bautista L Yali R Barja A. rgee: R Bindings for Calling the “Earth Engine” API. (2021). Available online at: https://CRAN.R-project.org/package=rgee

[53] Gorelick N Hancher M Dixon M Ilyushchenko S Thau D Moore R. Google Earth Engine: planetary-scale geospatial analysis for everyone. Remote Sens Environ. (2017) 202:18–27. 10.1016/j.rse.2017.06.031

[54] Wu Q. geemap: a Python package for interactive mapping with Google Earth Engine. J Open Source Softw. (2020) 5:2305. 10.21105/joss.02305

[55] NEX-DCP30. Ensemble Stats for NASA Earth Exchange Downscaled Climate Projections. Google Developers. Available online at: https://developers.google.com/earth-engine/datasets/catalog/NASA_NEX-DCP30_ENSEMBLE_STATS

[56] Taylor KE Stouffer RJ Meehl GA. An overview of CMIP5 and the experiment design. Bull Am Meteorol Soc. (2012) 93:485–98. 10.1175/BAMS-D-11-00094.135859545

[57] Meinshausen M Smith SJ Calvin K Daniel JS Kainuma MLT Lamarque JF. The RCP greenhouse gas concentrations and their extensions from 1765 to 2300. Climatic Change. (2011) 109:213. 10.1007/s10584-011-0156-z

[58] Parsons MA Duerr R Minster JB. Data citation and peer review. Eos Trans Am Geophys Union. (2010) 91:297–8. 10.1029/2010EO340001

[59] Arora P Boyne D Slater JJ Gupta A Brenner DR Druzdzel MJ. Bayesian networks for risk prediction using real-world data: a tool for precision medicine. Value Health. (2019) 22:439–45. 10.1016/j.jval.2019.01.006 30975395

[60] Bartram G Mahadevan S. Probabilistic prognosis with dynamic Bayesian networks. Int J Prognostics Health Manage. (2015) 6:4. 10.36001/ijphm.2015.v6i4.2290

[61] Dynamic Bayesian Networks to Assess Anthropogenic and Climatic Drivers of Saltwater Intrusion: A Decision Support Tool Toward Improved Management. Available online at: https://setac.onlinelibrary.wiley.com/doi/10.1002/ieam.4355 10.1002/ieam.4355 33034954

[62] Koski T Noble J. Bayesian Networks: An Introduction. West Sussex: John Wiley & Sons (2011). p. 340.

[63] Kratzer G Lewis FI Willi B Meli ML Boretti FS Hofmann-Lehmann R. Bayesian network modeling applied to feline calicivirus infection among cats in Switzerland. Front Vet Sci. (2020) 7:73. 10.3389/fvets.2020.00073 32175337 PMC7055399

[64] Lau CL Mayfield HJ Lowry JH Watson CH Kama M Nilles EJ. Unravelling infectious disease eco-epidemiology using Bayesian networks and scenario analysis: a case study of leptospirosis in Fiji. Environ Model Softw. (2017) 97:271–86. 10.1016/j.envsoft.2017.08.004

[65] Nguefack-Tsague G. Using Bayesian networks to model hierarchical relationships in epidemiological studies. Epidemiol Health. (2011) 33:e2011006. 10.4178/epih/e2011006 21779534 PMC3132659

[66] Pourret O Na¿m P Marcot B. Bayesian Networks: A Practical Guide to Applications. West Sussex: John Wiley & Sons (2008). p. 446.

[67] Qiu J Wang H Hu L Yang C Zhang T. Spatial transmission network construction of influenza-like illness using dynamic Bayesian network and vector-autoregressive moving average model. BMC Infect Dis. (2021) 21:164. 10.1186/s12879-021-05769-6 33568082 PMC7874476

[68] Zhang T Ma Y Xiao X Lin Y Zhang X Yin F. Dynamic Bayesian network in infectious diseases surveillance: a simulation study. Sci Rep. (2019) 9:10376. 10.1038/s41598-019-46737-0 31316113 PMC6637193

[69] Friedman N Geiger D Goldszmidt M. Bayesian network classifiers. Machine Learn. (1997) 29:131–63. 10.1023/A:1007465528199

[70] Best N Richardson S Thomson A. A comparison of Bayesian spatial models for disease mapping. Statistical Methods Med Res. (2005) 14:35–59. 10.1191/0962280205sm388oa 15690999

[71] Lawson A Lee D. Bayesian disease mapping for public health. In: Handbook of Statistics. Elsevier (2017). p. 443–81.

[72] Johnson GD. Small area mapping of prostate cancer incidence in New York State (USA) using fully Bayesian hierarchical modelling. Int J Health Geograph. (2004) 3:29. 10.1186/1476-072X-3-29 15588279 PMC544568

[73] Lahiri P Maiti T. Empirical bayes estimation of relative risks in disease mapping. Calcutta Statistical Assoc Bull. (2002) 53:213–24. 10.1177/0008068320020304

[74] Besag J York J Mollié A. Bayesian image restoration, with two applications in spatial statistics. Ann Inst Statistical Math. (1991) 43:1–20. 10.1007/BF00116466

[75] Alhdiri MAS Samat NA Mohamed Z. Disease mapping for stomach cancer in libya based on Besag-York-Mollié (BYM) model. Asian Pacific J Cancer Prev. (2017) 18:1479. 10.22034/APJCP.2017.18.6.1479 28669155 PMC6373820

[76] Knorr-Held L. Bayesian modelling of inseparable space-time variation in disease risk. Statistics Med. (2000) 19:2555–67. 10.1002/1097-0258(20000915/30)19:17/18<2555::AID-SIM587>3.0.CO;2-# 10960871

[77] Samat NA Mey LW. Malaria disease mapping in Malaysia based on Besag-York-Mollie (BYM) model. J Phys Confer Ser. (2017) 012167. 10.1088/1742-6596/890/1/012167

[78] Riebler A Sørbye SH Simpson D Rue H. An intuitive Bayesian spatial model for disease mapping that accounts for scaling. Stat Methods Med Res. (2016) 25:1145–65. 10.1177/0962280216660421 27566770

[79] Simpson DP Rue H Martins TG Riebler A Sørbye SH. Penalising model component complexity: a principled, practical approach to constructing priors. Preprint at: *arXiv*:14034630 (2015). Available online at: http://arxiv.org/abs/1403.4630

[80] Spiegelhalter DJ Best NG Carlin BP van der Linde A. The deviance information criterion: 12 years on. J R Statistical Soc Ser B. (2014) 76:485–93. 10.1111/rssb.12062

[81] Lustgarten JL Gopalakrishnan V Grover H Visweswaran S. Improving classification performance with discretization on biomedical datasets. AMIA Annu Symp Proc. (2008) 2008:445–9. 18999186 PMC2656082

[82] Dash R Paramguru R Dash R. Comparative analysis of supervised and unsupervised discretization techniques. Int J Adv Sci Technol. (2011) 2:3. Available online at: https://www.researchgate.net/publication/266058863_Comparative_Analysis_of_Supervised_and_Unsupervised_Discretization_Techniques25620721

[83] Gupta A Mehrotra KG Mohan C. A clustering-based discretization for supervised learning. Statistics Probability Lett. (2010) 80:816–24. 10.1016/j.spl.2010.01.015

[84] Cohen J. A coefficient of agreement for nominal scales. Educ Psychol Measure. (1960) 20:37–46. 10.1177/001316446002000104

[85] Akaike H. A new look at the statistical model identification. IEEE Trans Automatic Control. (1974) 19:716–23. 10.1109/TAC.1974.1100705

[86] Schwarz G. Estimating the dimension of a model. Ann Statistics. (1978) 6:461–4. 10.1214/aos/1176344136

[87] Blangiardo M Cameletti M Baio G Rue H. Spatial and spatio-temporal models with R-INLA. Spat Spatiotemporal Epidemiol. (2013) 7:39–55. 10.1016/j.sste.2013.07.003 24377114

[88] Ancel PY. Value of multinomial model in epidemiology: application to the comparison of risk factors for severely and moderately preterm births. Revue D'epidemiologie et de Sante Publique. (1999) 47:563–9. 10673590

[89] Cudjoe TKM Roth DL Szanton SL Wolff JL Boyd CM Thorpe RJ Jr. The epidemiology of social isolation: national health and aging trends study. J Gerontol Ser B. (2020) 75:107–13. 10.1093/geronb/gby037 29590462 PMC7179802

[90] Hedeker D. A mixed-effects multinomial logistic regression model. Statistics Med. (2003) 22:1433–46. 10.1002/sim.1522 12704607

[91] Jain RK Shukla R Singh P Kumar R. Epidemiology and risk factors for surgical site infections in patients requiring orthopedic surgery. Euro J Orthopaedic Surgery Traumatol. (2015) 25:251–4. 10.1007/s00590-014-1475-3 24806395

[92] Sadat-Hashemi SM Kazemnejad A Lucas C Badie K. Predicting the type of pregnancy using artificial neural networks and multinomial logistic regression: a comparison study. Neural Comput Appl. (2005) 14:198–202. 10.1007/s00521-004-0454-8

[93] Kuhn M. Classification and Regression Training. (2021). Available online at: https://github.com/topepo/caret

[94] Venables W Ripley B. Modern Applied Statistics with S. 4th ed. (2002). Available online at: http://www.stats.ox.ac.uk/pub/MASS4/

[95] Ho TK. Random decision forests. In: Proceedings of 3rd International Conference on Document Analysis and Recognition, Vol. 1. Montreal, QC (1995). p. 278–82.

[96] Hastie T Tibshirani R Friedman J. Elements of Statistical Learning: Data Mining, Inference, Prediction. 2nd ed. (2009). Available online at: https://web.stanford.edu/${sim}$hastie/ElemStatLearn/

[97] Haghighi M Johnson SB Qian X Lynch KF Vehik K Huang S. A comparison of rule-based analysis with regression methods in understanding the risk factors for study withdrawal in a pediatric study. Sci Rep. (2016) 6:1–12. 10.1038/srep30828 27561809 PMC5000469

[98] Hanson HA Martin C O'Neil B Leiser CL Mayer EN Smith KR. The relative importance of race compared to health care and social factors in predicting prostate cancer mortality: a random forest approach. J Urol. (2019) 202:1209–16. 10.1097/JU.0000000000000416 31246547 PMC8276188

[99] Kanerva N Kontto J Erkkola M Nevalainen J Männistö S. Suitability of random forest analysis for epidemiological research: exploring sociodemographic and lifestyle-related risk factors of overweight in a cross-sectional design. Scand J Public Health. (2018) 46:557–64. 10.1177/1403494817736944 29082809

[100] Kuroki Y. Risk factors for suicidal behaviors among Filipino Americans: a data mining approach. Am J Orthopsychiatry. (2015) 85:34. 10.1037/ort0000018 25110976

[101] Puterman E Weiss J Hives BA Gemmill A Karasek D Mendes WB. Predicting mortality from 57 economic, behavioral, social, and psychological factors. Proc Natl Acad Sci USA. (2020) 117:16273–82. 10.1073/pnas.1918455117 32571904 PMC7369318

[102] Tao D Sun J Wu X Li X Shen J Maybank SJ. Probabilistic tensor analysis with Akaike and Bayesian information criteria. In: Ishikawa M Doya K Miyamoto H Yamakawa T, editors. Neural Information Processing, Lecture Notes in Computer Science. Berlin: Springer (2008). p. 791–801.

[103] Zhang X Tang F Ji J Han W Lu P. Risk prediction of dyslipidemia for Chinese Han adults using random Forest survival model. Clin Epidemiol. (2019) 11:1047. 10.2147/CLEP.S223694 31849535 PMC6911320

[104] Muhammad LJ Algehyne EA Usman SS Ahmad A Chakraborty C Mohammed IA. Supervised machine learning models for prediction of COVID-19 infection using epidemiology dataset. SN Comput Sci. (2020) 2:11. 10.1007/s42979-020-00394-7 33263111 PMC7694891

[105] Brieman L Cutler Ad. Random Forests. (2020). Available online at: https://www.stat.berkeley.edu/\simbreiman/RandomForests/

[106] Li X Lord D Zhang Y Xie Y. Predicting motor vehicle crashes using support vector machine models. Accident Anal Prev. (2008) 40:1611–8. 10.1016/j.aap.2008.04.010 18606297

[107] Min JH Lee YC. Bankruptcy prediction using support vector machine with optimal choice of kernel function parameters. Expert Syst Appl. (2005) 28:603–14. 10.1016/j.eswa.2004.12.008

[108] Wang W Men C Lu W. Online prediction model based on support vector machine. Neurocomput. (2008) 71:550–8. 10.1016/j.neucom.2007.07.020

[109] Yang H Chan L King I. Support vector machine regression for volatile stock market prediction. In: International Conference on Intelligent Data Engineering and Automated Learning. Berlin; Heidelberg: Springer (2002) p. 391–6.

[110] Meyer D Dimitriadou E Hornik K Weingessel A Leisch F C++-code) CCC, (libsvm, et al. e1071: Misc Functions of the Department of Statistics, Probability Theory Group (Formerly: E1071). TU Wien. (2021). Available online at: https://CRAN.R-project.org/package=e1071

[111] Bafjaish SS. Comparative analysis of naive Bayesian techniques in health-related for classification task. J Soft Comput Data Mining. (2020) 1:1–10.

[112] Kuyo M Mwalili S Okang'o E. Machine learning approaches for classifying the distribution of COVID-19 sentiments. Open J Statistics. (2021) 11:620–32. 10.4236/ojs.2021.115037

[113] Mutuvi S Doucet A Lejeune G Odeo M. A dataset for multi-lingual epidemiological event extraction. In: Proceedings of the 12th Conference on Language Resources and Evaluation (LREC 2020). Marseille (2020). p. 4139–44. Available online at: https://hal.archives-ouvertes.fr/hal-02732848

[114] Nasir A Shah MA Ashraf U Khan A Jeon G. An intelligent framework to predict socioeconomic impacts of COVID-19 and public sentiments. Comput Electrical Eng. (2021) 96:107526. 10.1016/j.compeleceng.2021.107526 34658455 PMC8502699

[115] Jensen FV. Gradient descent training of Bayesian networks. In:Hunter A Parsons S, editors. Symbolic and Quantitative Approaches to Reasoning and Uncertainty, Lecture Notes in Computer Science. Berlin: Springer (1999). p. 190–200.

[116] Lee TJ Goldszmidt M. TAN Tree Augmented Naive-Bayes Bayesian Network Classifier Version 2.1 User Manual (1998). p. 1–27. Available online at: http://edi.erg.sri.com/tan/TANintro.htm

[117] Tsamardinos I Brown LE Aliferis CF. The max-min hill-climbing Bayesian network structure learning algorithm. Mach Learn. (2006) 65:31–78. 10.1007/s10994-006-6889-7

[118] Zeng Y Luo J Lin S. Classification using Markov blanket for feature selection. In: 2009 IEEE International Conference on Granular Computing. Nanchung (2009). p. 743–7. 10.30880/jscdm.2020.01.02.001

[119] Pearl J. Causality: Models, Reasoning, and Inference. Cambridge: Cambridge University Press (2000). p. 412.

[120] Burnham KP Anderson DR editors. Information and likelihood theory: a basis for model selection and inference. In: Burnham KP Anderson DR, editors. Model Selection and Multimodel Inference: A Practical Information-Theoretic Approach. New York, NY: Springer (2002). p. 49–97.

[121] de Campos CP Ji Q. Efficient structure learning of Bayesian networks using constraints. J Mach Learn Res. (2011) 12:663–689.

[122] Salnikov V Schaub MT Lambiotte R. Using higher-order Markov models to reveal flow-based communities in networks. Sci Rep. (2016) 6:23194. 10.1038/srep23194 27029508 PMC4814833

[123] Fernandes R. dbnlearn: Dynamic Bayesian Network Structure Learning, Parameter Learning Forecasting. (2020). Available online at: https://CRAN.R-project.org/package=dbnlearn

[124] Biggs EN Maloney PM Rung AL Peters ES Robinson WT. The relationship between social vulnerability and COVID-19 incidence among louisiana census tracts. Front Public Health. (2021) 8:617976. 10.3389/fpubh.2020.617976 33553098 PMC7856141

[125] Huang Q Jackson S Derakhshan S Lee L Pham E Jackson A. Urban-rural differences in COVID-19 exposures and outcomes in the South: a preliminary analysis of South Carolina. PLoS ONE. (2021) 16:e0246548. 10.1371/journal.pone.0246548 33534870 PMC7857563

[126] Karaye IM Horney JA. The impact of social vulnerability on COVID-19 in the US: an analysis of spatially varying relationships. Am J Prev Med. (2020) 59:317–25. 10.1016/j.amepre.2020.06.006 32703701 PMC7318979

[127] Oates GR Juarez LD Horswell R Chu S Miele L Fouad MN. The association between neighborhood social vulnerability and COVID-19 testing, positivity, and incidence in Alabama and Louisiana. J Commun Health. (2021) 46:1115-23. 10.1007/s10900-021-00998-x 33966116 PMC8106900

[128] Brennan J Bannick MS Kassebaum N Wilner LB Thomson A Aravkin A. Analysis and methods to mitigate effects of under-reporting in count data. arXiv. (2021). 10.48550/arXiv.2109.12247

[129] Oliveira ACS de Morita LHM Silva EB Granzotto D Zardo LAR Fontes C. Bayesian modeling of COVID-19 cases with a correction to account for under-reported cases. Infect Dis Model. (2020). 10.1101/2020.05.24.20112029 32995681 PMC7513875

[130] Stoner O Economou T. Multivariate hierarchical frameworks for modeling delayed reporting in count data. Biometrics. (2019). 10.1111/biom.13188 31737902 PMC7540263

[131] Research and High Performance Computing (2020). Available online at: https://kb.iu.edu/d/apes

